# Driven progressive evolution of genome sequence complexity in Cyanobacteria

**DOI:** 10.1038/s41598-020-76014-4

**Published:** 2020-11-04

**Authors:** Andrés Moya, José L. Oliver, Miguel Verdú, Luis Delaye, Vicente Arnau, Pedro Bernaola-Galván, Rebeca de la Fuente, Wladimiro Díaz, Cristina Gómez-Martín, Francisco M. González, Amparo Latorre, Ricardo Lebrón, Ramón Román-Roldán

**Affiliations:** 1 Institute of Integrative Systems Biology (I2Sysbio), University of València and Consejo Superior de Investigaciones Científicas (CSIC), 46980 Valencia, Spain; 2 Foundation for the Promotion of Sanitary and Biomedical Research of Valencian Community (FISABIO), 46020 Valencia, Spain; 3 CIBER in Epidemiology and Public Health, 28029 Madrid, Spain; 4 Department of Genetics, Faculty of Sciences, University of Granada, 18071 Granada, Spain; 5 Laboratory of Bioinformatics, Institute of Biotechnology, Center of Biomedical Research, 18100 Granada, Spain; 6 Centro de Investigaciones sobre Desertificación, Consejo Superior de Investigaciones Científicas (CSIC), University of València and Generalitat Valenciana, 46113 Valencia, Spain; 7 Department of Genetic Engineering, CINVESTAV, 36821 Irapuato, Mexico; 8 Department of Applied Physics II and Institute Carlos I for Theoretical and Computational Physics, University of Málaga, 29071 Málaga, Spain; 9 Institute for Cross-Disciplinary Physics and Complex Systems (IFISC), Consejo Superior de Investigaciones Científicas (CSIC) and University of Balearic Islands, 07122 Palma de Mallorca, Spain; 10 Department of Applied Physics, University of Granada, 18071 Granada, Spain

**Keywords:** Computational biology and bioinformatics, Evolution, Genetics, Microbiology

## Abstract

Progressive evolution, or the tendency towards increasing complexity, is a controversial issue in biology, which resolution entails a proper measurement of complexity. Genomes are the best entities to address this challenge, as they encode the historical information of a species’ biotic and environmental interactions. As a case study, we have measured genome sequence complexity in the ancient phylum Cyanobacteria. To arrive at an appropriate measure of genome sequence complexity, we have chosen metrics that do not decipher biological functionality but that show strong phylogenetic signal. Using a ridge regression of those metrics against root-to-tip distance, we detected positive trends towards higher complexity in three of them. Lastly, we applied three standard tests to detect if progressive evolution is passive or driven—the minimum, ancestor–descendant, and sub-clade tests. These results provide evidence for driven progressive evolution at the genome-level in the phylum Cyanobacteria.

Treatises on biological evolution reflect a conflict between the relative roles played by contingency and necessity^1^. An important tradition in evolutionary biology, based on a large amount of empirical evidence, considers that contingency marks the dynamics of evolution in a way that makes it unpredictable^1–3^. The trend towards the appearance of increasing complexity falls within the frame of contingent evolution insofar as it is inevitable given that, passively, we can expect that sooner or later more complex entities will evolve from the original, simpler entities. This is what Gould^2^ referred to as ‘the passive tendency towards complexity marked by the minimum initial complexity wall’.

A central task for those studying complexity is to provide an accurate measure to ascertain if there is a trend of increasing complexity^3,4^. In fact, a necessary condition for progressive and open-ended evolution is to prove the existence of a metric that increases with the evolutionary age of the corresponding organisms^4,5^. We suggest that we can find such metrics in the genomes^6^. Genomes probably provide the best record of the biological history of a species. Not only do they enable us to reconstruct their phylogenetic relationships but they also contain information gained from their continuous biotic and environmental interactions over time^6–8^. Standard genome parameters such as genome size, number of genes, and gene components (i.e., introns, exons) are insufficient indicators of genome complexity because they partially capture the historical information encoded in a genome^9,10^. We suggest here that metrics unassociated with biological functions may improve our measurements of genome sequence complexity. However, some metrics that have been previously applied to genomes are too broad, and not all of them accurately capture all the necessary information gleaned from a genome during its evolutionary history^6,11^. For example, algorithmic complexity^12,13^ is inconveniently maximized for randomness and the effective complexity of Gell-Mann and Lloyd^14^ is recommended for collections or ensembles of sequences, but in several cases such as that seen in genome sequences, it is not clear how to define an appropriate ensemble. Likewise, those metrics based on mutual information or statistical dependence^15,16^ also quantify the complexity of sequence ensembles rather than the complexity of a single sequence.

Here we consider six metrics that are calculated on individual genomes. The first four metrics are based on the Sequence Compositional Complexity (*SCC*) derived from a four-symbol DNA sequence or the binary sequences resulting from grouping the four nucleotides into S(C,G) versus W(A,T) or R(A,G) versus Y (T,C), or K(A,C) versus M(T,G), thus obtaining *SCC*_*SW*_, *SCC*_*RY*_ and *SCC*_*KM*_ metrics, respectively^17^. These four metrics increase with the number of parts (i.e. compositional domains) as well as the length and compositional differences among them found in a genome sequence by a segmentation algorithm. These metrics parallel the concept of ‘pure complexity’ of McShea^18^ and McShea and Brandon^3^, where complexity is more closely related to the number of part types of an individual than with the number of functions.

The fifth metric we used is the Biobit (*BB*), a metric based on the difference between the maximum entropy for a *k*-*mer* of a random genome of the same length as the genome under consideration and the entropy of that genome for such a *k*-mer^19^. Lastly, we used the Genomic Signature (*GS*), also a *k*-*mer*-based metric, which is the value corresponding to the *k*-*mer* that maximizes the difference between observed and expected equi-frequent classes of *mers*. *GS* is based on the relative abundances of short oligonucleotides^20^ and chaos game representation applied to genomes^21,22^.

We tested the above-mentioned metrics by analyzing the genome evolution of an ancient and diverse group of organisms: the phylum Cyanobacteria. These microorganisms played a fundamental role in the evolution of life on Earth. The fossil record shows that they were here 2.0 Billion years ago (Bya) and molecular clock analyses indicate that the phylum originated over 2.5 Bya^23,24^. By releasing oxygen through photosynthesis, Cyanobacteria caused the Great Oxidation Event about 2.3 Bya and changed the history of life on Earth^25^. The oxidation of the environment allowed the evolution of complex multicellular life forms^26^, leading to the origin of eukaryotic crown groups including plants and animals^27^. As it is well known, Cyanobacteria also were the progenitor of plastids through symbiosis with ancient eukaryotes^28^.

Cyanobacteria are morphologically diverse. The group was traditionally classified into five subsections according to several biological criteria^29,30^. Unicellular cyanobacteria are classified in subsections I and II, depending on their mode of reproduction. Those from subsection I (Chroococcales) divide only by binary fission while those from subsection II (Pleurocapsales) are capable of reproducing also by multiple fission giving rise to small cells called baeocytes. Filamentous cyanobacteria are classified into subsections III, IV, and V. Those from subsection III (Oscillatoriales) are composed only by vegetative cells that reproduce by intercalary division. Cyanobacteria from subsections IV and V are capable of cell differentiation producing trichomes composed of vegetative cells and heterocysts for nitrogen fixation. In addition, some members also produce hormogonia for dispersal and akinetes for dormancy. Members of subsection IV (Nostocales) always divide in a plane at right angles to the long axis of the trichome; while those from subsection V (Stigonematales) may also divide at parallel axes relative to the long axes of the trichome.

Of the above subsections of Cyanobacteria, only Stigonematales are monophyletic^24,31^. More recent classification schemes using phylogenetic analysis from 31 conserved protein sequences divide Cyanobacteria into nine different groups^32^. These include Gleobacterales, Synechococcales, Oscillatoriales, Chroococcales, Pleurocapsales, Spirulinales, Rubidibacter/Halothece, Chroococcidiopsidales, and Nostocales. Of these groups, Chroococcales, Oscillatoriales, and Synechococcales are not monophyletic. This classification scheme attempts to reconcile phylogenetic history with several aspects of morphology and cytology. Other phylogenetic analyses based on 31 concatenated conserved proteins divide cyanobacteria into seven groups^33^. These groups are named from A to G (groups B and C are further subdivided into B1, B2 and B3 and C1, C2 and C3) and all of them are monophyletic. Furthermore, taxon addition and subtraction analyses on a concatenated dataset of 137 conserved proteins and two rRNA sequences, allowed the identification of long-branch attraction artifacts^34^. The resulting tree was used to classify cyanobacteria into 6 monophyletic groups, corresponding to some of the A to G lineages. Finally, phylogenetic analysis on a concatenated dataset of 43 proteins from 208 taxa, recovered all A–G groups and revealed the existence of novel monophyletic lineages located at the base of the tree^35^. Clearly, the taxonomy and evolution of Cyanobacteria are active areas of research. The classification of Cyanobacteria is likely to change in the near future as more lineages are sequenced and analyzed.

In this study, we test whether there is a statistically and phylogenetically supported driven tendency towards increasing genome sequence complexity in the evolution of Cyanobacteria as reflected by some of their metrics of genomic complexity.

## Results

### Complexity measures throughout Cyanobacteria phylogeny

**Figure 1:**
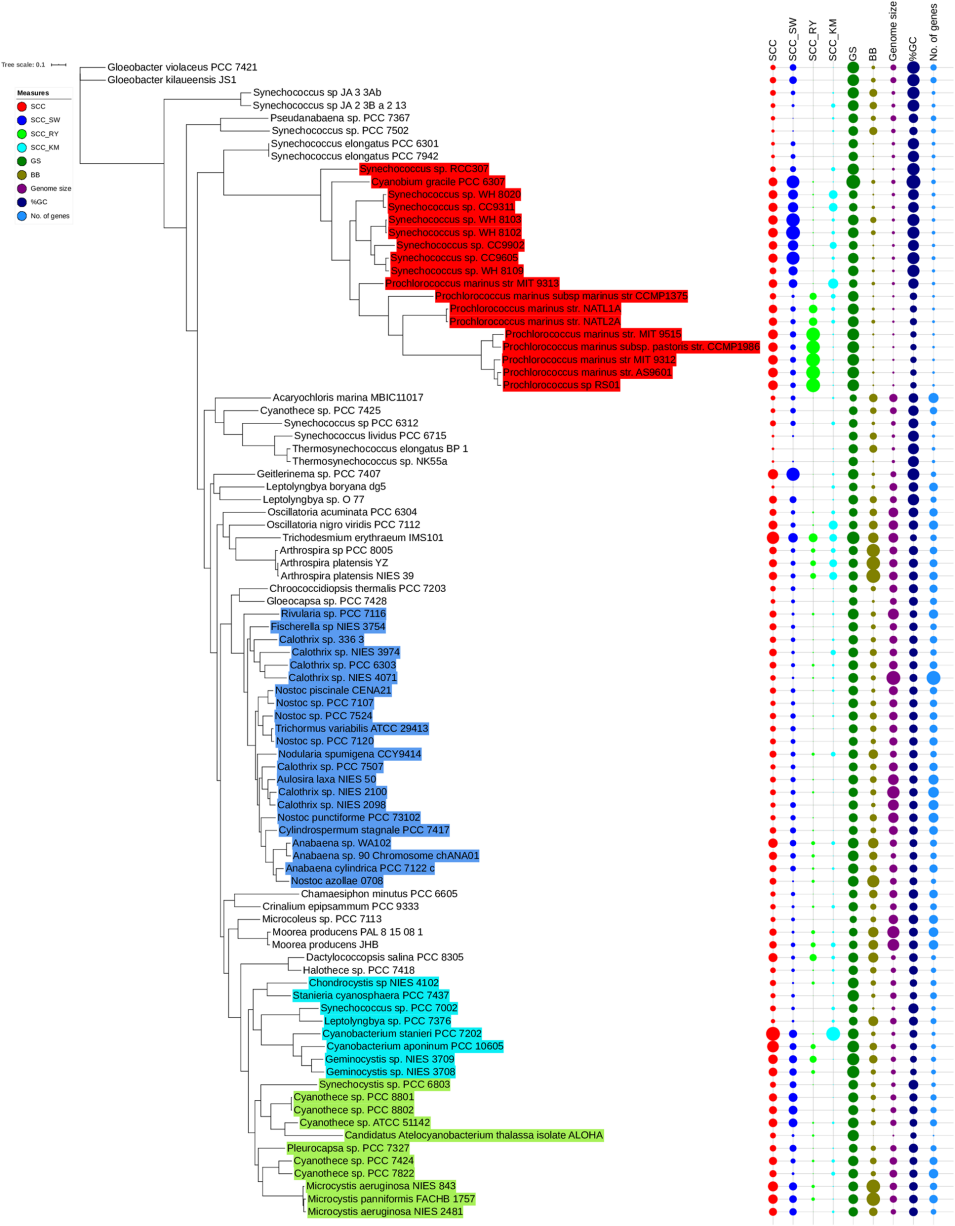
Phylogeny of Cyanobacteria with the metrics of sequence complexity and genome parameters for each chosen genome. The values of metrics and parameters are proportional to circle size. The colored species correspond to four monophyletic sub-clades that were used to test evidence of a driven trend for each sub-clade (see also Fig. S2).

The values of the four *SCCs*, *BB* and *GS* metrics as well as three standard genome parameters (Genome size, %GC and No. of genes) (see “Methods” section) for 91 Cyanobacterial genomes are reported in Table S1. Figure 1 shows a maximum likelihood phylogeny of Cyanobacteria whose branch lengths are proportional to the number of amino acid substitutions (see “Methods” section). The phylogeny is in general agreement with the previous analyses^24,31,32^.

### Phylogenetic signal

All metrics of complexity and genome parameters showed a significant phylogenetic signal (Table 1), indicating that for all cases genomes of closely related cyanobacterial species tend to resemble more than two randomly selected genomes (Fig. 1). However, the magnitude of the phylogenetic signal differs across metrics and parameters, with %GC and *GS* showing the highest values, which probably reflects different forces shaping the evolution of all these metrics and parameters (see “Discussion” section).

**Table 1:** Phylogenetic signals (*K*) of metrics of genome sequence complexity and genome parameters.

Metrics of genome sequence complexity and genome parameters	*K*	Probability, *P*
*SSC*	0.34	0.001
*SCC*_*RY*_	0.66	0.001
*SCC*_*SW*_	0.32	0.001
*SCC*_*KM*_	0.26	0.001
*BB*	0.15	0.001
*GS*	1.00	0.001
Genome size	0.46	0.001
%GC	3.96	0.001
No. of genes	0.31	0.001

The first six rows correspond to the metrics and the last three to genome parameters.

### Phylogenetic correlations

To gain a better understanding of the metrics, after we corrected the phylogenetic signals, we evaluated how they correlate with each other and, particularly, with the genomic parameters (Table 2). It is worth noticing that two metrics, *SCC* and *SCC*_*RY*_ correlate with other metrics and parameters (six correlations each one), accounting for 43% of all significant correlations.

**Table 2:** Phylogenetic Pearson correlations (*r*) among metrics of genome complexity and genome parameters.

	*SCC*	*SCC*_*SW*_	*SCC*_*RY*_	*SCC*_*KM*_	*BB*	*GS*	Genome Size	%GC
*SCC*_*SW*_	0.66***							
*SCC*_*RY*_	0.52***	0.09^ns^						
*SCC*_*KM*_	0.30**	0.09^ns^	0.03^ns^					
*BB*	0.38***	− 0.02^ns^	0.53***	− 0.04^ns^				
*GS*	0.34***	0.20^ns^	0.41***	− 0.20^ns^	0.19^ns^			
Genome Size	0.22*	0.10^ns^	0.31**	− 0.05^ns^	0.32**	0.001^ns^		
%GC	− 0.06^ns^	0.26*	− 0.38***	− 0.11^ns^	− 0.09^ns^	− 0.1^ns^	− 0.11^ns^	
No. of genes	0.12^ns^	0.07^ns^	0.26*	− 0.09^ns^	0.26*	− 0.09^ns^	0.86***	− 0.09^ns^

Statistical significance was corrected by false discovery rates to control for multiple testing.

***P < 0.001; **0.001 < *P* < 0.01; *0.01 < *P* < 0.05; ^ns^*P* > 0.05.

### Ridge regression of complexity metrics versus distance from the root

**Figure 2:**
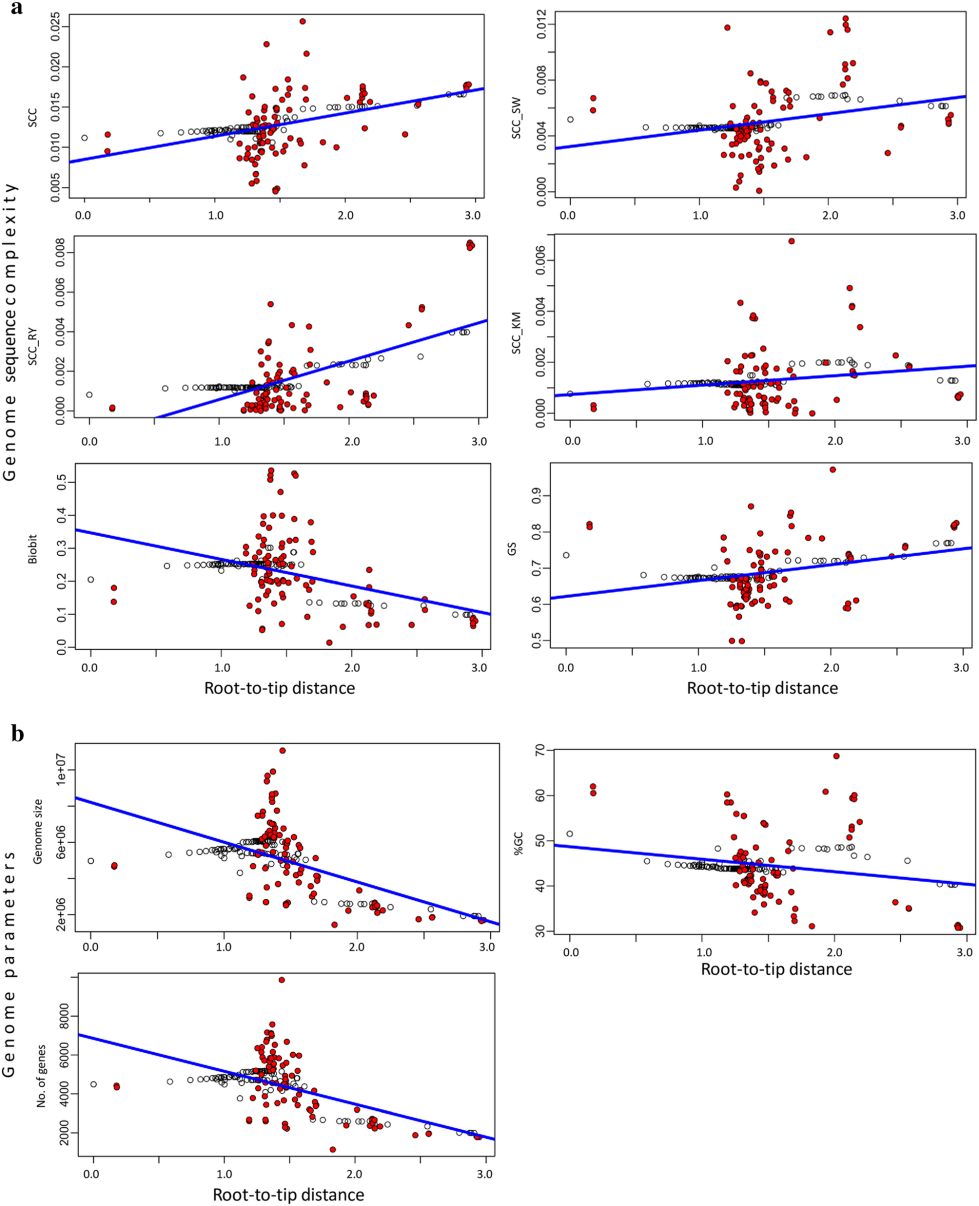
Phylogenetic trends of genomic complexity metrics (**a**) and standard genome parameters (**b**). The estimated genomic value for each tip (red circles) or node (white circles) in the phylogenetic tree is regressed against its evolutionary age (i.e., distance from the root). The statistical significance of the regression is tested against 10,000 slopes obtained under simulated Brownian evolution. The slopes and their *P* values are shown in Table S2.

Using ridge regression of genome complexity metrics and genome parameters versus distance from the root, we have studied whether there is evidence of evolutionary trends, having detected interesting patterns (Fig. 2). Of the complexity metrics, four out of six show a statistically significant positive trend (*SCC*,* SSC*_*SW*_,* SCC*_*RY*_ and *GS*), indicating that complexity, as determined by our proposed criteria, has increased with the distance from the root. In contrast, *SCC*_*KM*_ shows no trend and *BB* reveals a significant negative trend. Interestingly, genome parameters show no evidence of any evolutionary trend.

### Driven trends in Cyanobacteria

A critical question regarding trends is if they are passive or driven. To evaluate this, we have applied three types of tests (see “Methods” section for a detailed description): the minimum (with three types of proofs), the ancestor–descendant, and the subclade (with two types of proofs) tests.

**Figure 3:**
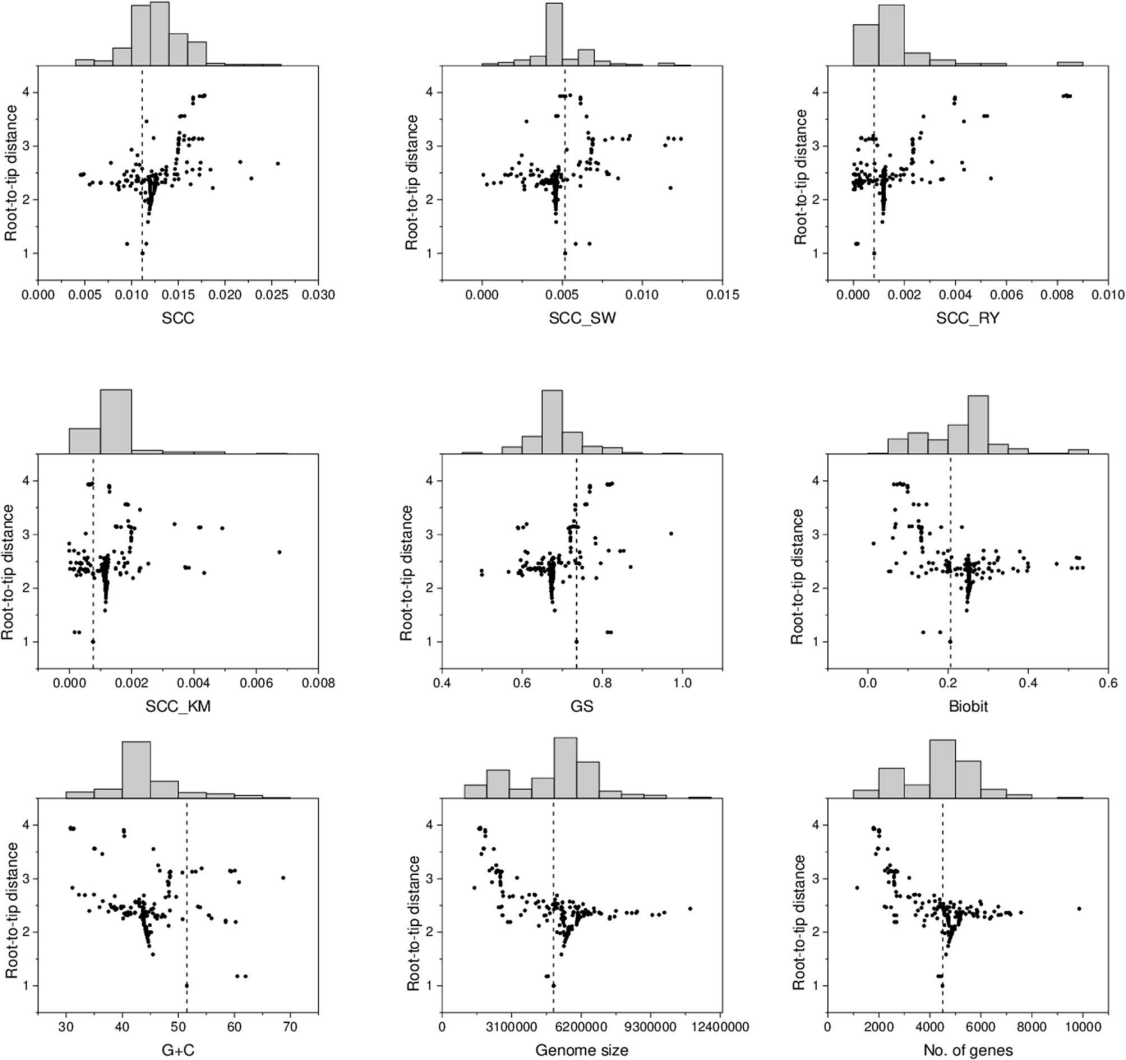
Distribution of metrics and parameters according to root-to-tip distance. The interior dashed line corresponds to the value of the basal clade, *x*_*b*_. The histograms that appear above each figure correspond to the number of accumulated values of metrics and parameters (regardless of the age) ranging from lower (left) to higher (right) values than *x*_*b*_.

Regarding the first proof of the minimum test (i.e., skewness), we observed that the skewness of all metrics (except *SCC* and *GC* content) for the entire phylum exhibit significant and positive skewness (D’Agostino–Pearson test, *n* = 91; Table 3), which supports a left wall for these metrics and parameters that is compatible with either a passive or a driven trend. Nevertheless, it is expected that if the minimum value of a given metric or parameter increases with evolutionary time, then the trend will probably be driven. To test this we have taken as the minimum the estimated value of the most basal clade, *x*_*b*_, for each metric/parameter (Fig. 1). However, as it can be observed (Fig. 3), there are lower or higher values than the one corresponding to the basal clade for any metric/parameter. Then, it is necessary to study in greater depth the distribution of lower and higher values with respect to *x*_*b*_ in order to have evidence of the putative existence of a driven trend. With this end, we carried out the second proof of the minimum test, where we measure |*x*_*d*_* − x*_*b*_|, the absolute difference between descendants’ clades and the most basal clade. Table 3 shows whether there is a statistical difference (Chi-square test) between those clades (179 in total) that are higher or lower than the basal clade, *x*_*b*_. As it can be observed, all the tests are significant with four metrics (*SCC*, *SCC*_*RY*_, *SCC*_*KM*_ and *BB*) and two parameters (Genome size and No. of genes) showing a significant increase in the metric/parameter with respect to the corresponding basal values. In contrast, two metrics (*SCC*_*SW*_ and *GS*) and one parameter (%GC) present a significant decrease. Finally, employing a Student’s *t*-test (third proof of the minimum test), we tested if there is a statistical difference between the average value of the absolute difference (|*x*_*d*_ *− x*_*b*_|) of a given metric or parameter higher or lower than *x*_*b*_. It can be observed (Table 3) that three metrics (*SCC*_*SW*_, *SCC*_*RY*_ and *SCC*_*KM*_) show a statistical difference in favor of a higher value than *x*_*b*_ and one metric (*GS*) and the three parameters (Genome size, %GC content, and No. of genes) present a statistical difference in favor of a lower value than *x*_*b*_.

**Table 3:** Proofs of the minimum test.

Complexity measure	Skewness	*P* value	Higher than *x*_*b*_		Lower than *x*_*b*_		Chi-square test *P* value	Student’s *t-*test *P* value
			*n*	|*x*_*d*_* − x*_*b*_|	*n*	|*x*_*d*_* − x*_*b*_|		
*SCC*	0.3470	2.78E*−*01	139	0.00265	40	0.00207	1.3659E*−*13	0.0848
*SCC*_*SW*_	0.9455	5.31E*−*04	48	0.00215	131	0.00108	5.5147E*−*10	5.8066E*−*07
*SCC*_*RY*_	2.1530	9.49E*−*12	130	0.00115	49	0.00051	1.1410E*−*09	0.0031
*SCC*_*KM*_	1.9214	6.76E*−*11	138	0.00079	43	0.00035	1.6496E*−*12	0.0005
*BB*	0.7018	2.31E*−*02	116	0.07421	63	0.07290	7.4510E*−*05	0.4452
*GS*	0.6050	4.30E*−*02	30	0.05647	149	0.07073	5.8695E*−*19	0.0460
Genome Size	0.3805	2.31E*−*01	112	1,117,595	67	1,959,615	0.0008	3.3185E*−*07
%GC	0.6496	4.53E*−*02	20	6.245	159	8.705	2.7724E*−*25	0.0053
No. of genes	0.3367	3.78E*−*01	105	796.8	74	1488.6	0.0205	1.1460E*−*07

D’Agostino–Pearson test of skewness for the entire phylum (*n* = 91), number (*n*) of times that the metric/parameter of a given derived internal or terminal node (*x*_*d*_) is higher or lower than the basal node (*x*_*b*_) (Chi-square test), as well as the average absolute difference (|*x*_*d*_* − x*_*b*_|, Student’s *t*-test) between nodes that are higher or lower than *x*_*b*_. The first six rows correspond to the metrics and the last three to genome parameters.

**Figure 4:**
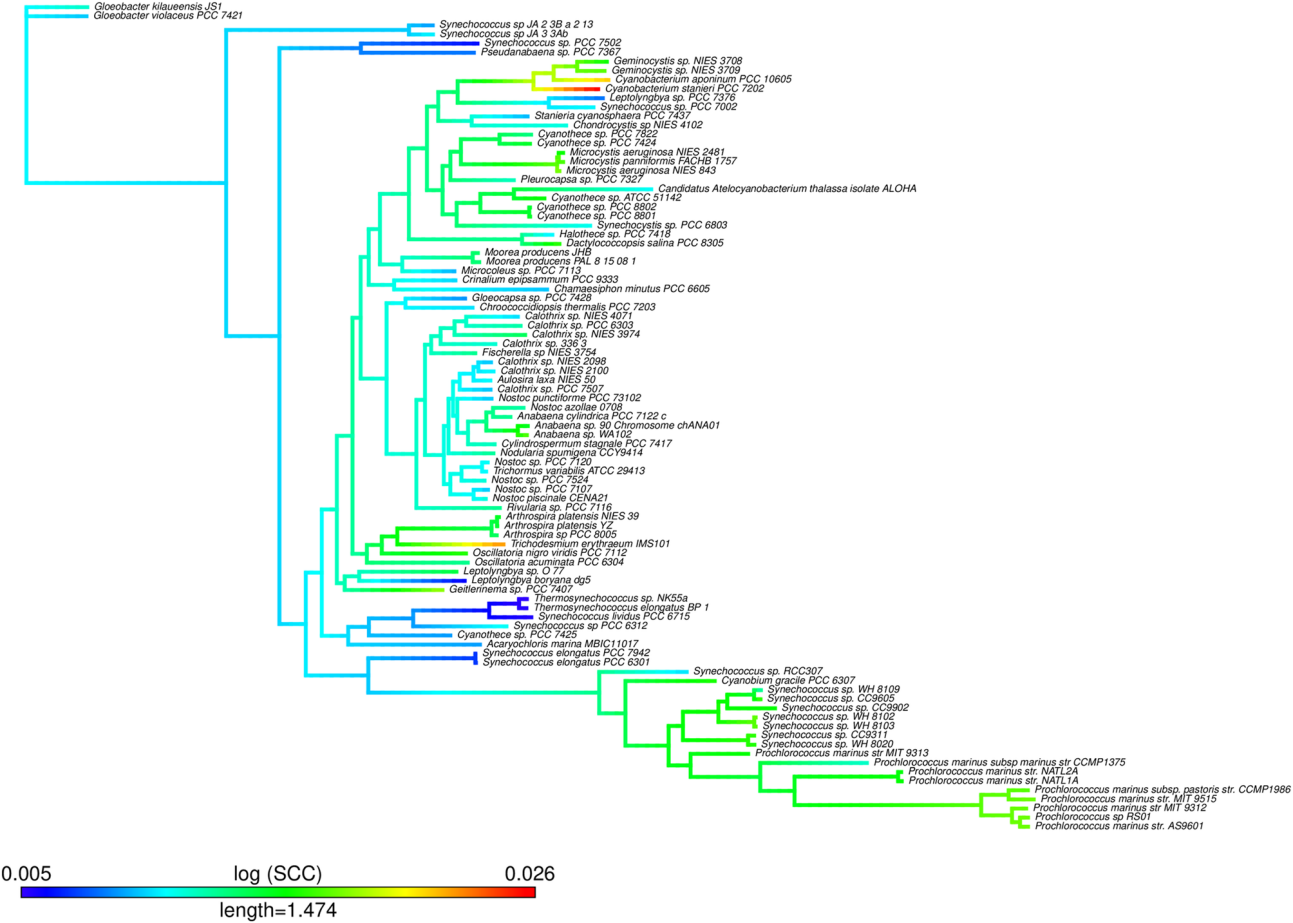
Mapping of the *SCC* complexity metric on the Cyanobacteria tree.

Regarding the ancestor–descendant test (see “Methods” section for a detailed description) we tabulated the derived clades for all possible nodes and whether they present a higher, lower, or equal value of the metric/parameter than the ancestral clade corresponding to each node. In order to avoid bias due to proximity to the putative left wall, McShea^36^ recommended applying the test only to those clades where both ancestor and descendent are higher than the average value of the metric/parameter. As it can be observed (Fisher exact test, Table 4) this exigent test shows that metrics *SCC* and *GS* and the three genome parameters are in favor of a driven trend. A good visualization of the ancestor–descendant proof on the phylogeny of the Cyanobacteria for each metric/parameter has been obtained by estimating the values of internal nodes using a maximum likelihood function and interpolating the value along each edge (see “Methods” section). Figure 4 shows the mapping corresponding to the *SCC* metric where the driven positive trend of this metric can be clearly appreciated (Fig. S1 for the mapping of the rest of metrics/parameters).

**Table 4:** Ancestor–descendant test.

Complexity measure	Derived nodes with a higher value than the ancestor of a given clade	Derived nodes with a lower value than the ancestor of a given clade	Fisher exact text *P* value
*SCC*	36	2	0.0001
*SCC*_*SW*_	19	9	0.2772
*SCC*_*RY*_	15	5	0.1908
*SCC*_*KM*_	15	15	1.0000
*BB*	58	32	0.0703
*GS*	33	5	0.0011
Genome Size	68	36	0.0018
%GC	68	8	0.0350
No. of genes	38	32	0.0143

For any complexity metric/genome parameter, we test whether the derived clades present higher or lower values than the corresponding ancestral clade for any node. The first six rows correspond to the metrics and the last three to genome parameters.

Finally, the last test applied is the sub-clade test, with the two associated proofs. In the first proof, we tested whether the trend observed at the phylum level is also observed in four selected monophyletic clades and second, we have also applied the skewness test to either the entire phylum (results are given in Table 3) and to the chosen sub-clades. We have chosen four monophyletic clades formed by clusters 97, 132, 162, and 172 that harbor 18, 22, 11, and 8 species, respectively (four-colors in Fig. 1 and Fig. S2). Clade 97 is formed by Synechococcus, Prochlorococcus, and Cyanobium; clade 132 corresponds to Nostocales (subsections IV and V); clade 162 contains Cyanothece and Microcystis; and clade 172, among others, contains Geminocystis and Cyanobacterium. The most relevant result found was that some metrics of genome complexity show statistically significant positive trends (*SCC*, *SCC*_*RY*_, and *GS*) and some others show negative trends (*SCC*_*SW*_ and *SCC*_*KM*_), whereas the genome parameters do not show any positive trends (Table S2; Fig. S3). Thus, we keep *SCC*, *SCC*_*RY*_ and *GS* as the metrics showing positive trends at both levels of phylogenetic resolution.

Regarding the second proof for the sub-clade test, we have examined if the monophyletic sub-clade drawn from the right tail of the entire distribution should have a statistically significant average higher value than the one corresponding to the entire phylum. Regarding the skewness of the phylum (Table 3), we observe that all metrics (except *SCC* and %GC) exhibit significant and positive skewness. However, this test of skewness cannot be applied to the four chosen monophyletic sub-clades either because (a) the average value (median) of a given metric/parameter for each sub-clade was lower than the median of the phylum (16 cases out of 36) or, (b) there was no statistical evidence (the remaining 20 cases) of a higher median (Mood’s median test) of a given metric/parameter for each sub-clade than the median of the entire phylum (see Table S3).

In summary**,** the overall results obtained in relation to the evidence found for a trend in a given metric or parameter, i.e., the phylogenetic signal, the number of significant correlations against the rest of metrics/parameters, as well as whether the trend is driven or not (Table 5) show that *SCC*, *SCC*_*RY*_ and to a lower extent *GS* present the highest scores, and can thus be considered metrics evidencing progressive evolution of Cyanobacteria.

**Table 5:** Summary of the results for each sequence complexity metric and genome parameter.

Sequence complexity metric/parameter	*K*	Number of significant correlations	General trend	Driven trend						
				Minimum test			Ancestor–descendant test	Trend in the four sub-clades		
				Skewness	Chi-square test	Student’s *t*-test		+	−	0
*SCC*	+	6	+	0	+	0	+	2	0	2
*SCC*_*SW*_	+	2	+	+	−	+	0	1	2	1
*SCC*_*RY*_	+	6	+	+	+	+	0	2	0	2
*SCC*_*KM*_	+	1	0	+	+	+	0	0	2	2
*BB*	+	4	−	+	+	0	0	0	1	3
*GS*	+	2	+	+	−	−	+	3	0	1
Genome Size	+	4	−	+	+	−	+	0	2	2
%GC	+	2	−	0	−	−	+	0	2	2
No. of genes	+	3	−	+	+	−	+	0	2	2

*K* is the phylogenetic signal. The signs “ + ”, “−” or “0” indicate the existence of a positive, negative or no statistical evidence, respectively, for the corresponding test: the general trend, the driven trend, the three types of proof of the minimum (i.e., skewness, Chi-square test and Student’s *t*-test), the ancestor–descendant test and the trend in the case of the four sub-clades. The first six rows correspond to the metrics and the last three to genome parameters.

## Discussion

Genomes probably provide the best record of the biological history of species. Not only do they enable us to reconstruct their phylogenetic relationships but they also contain information gained from their continuous biotic and environmental interactions over time^6,8^. This information is an elusive but crucial component of the genome, whose study as a whole deserves deeper attention because it holds clues to answer many biological questions, particularly those of an evolutionary nature.

The genome has distinct layers of information encoded in DNA sequences^10,37^. The most well-known are those involved in biological function, such as the typical genome division into coding and non-coding parts or the differential conservation shown by distinct codon positions due to the differential evolutionary constraints acting within genes^38–40^. In the present study, we intend to capture or approximate the genome information held in these layers using certain metrics (collectively named ‘genome complexity metrics’) to determine whether they show phylogenetic signals and indicate some kind of evolutionary trend. To do so, we use a group of organisms with a long phylogenetic history: the phylum Cyanobacteria. *SCC* accounts for the global compositional complexity of a DNA sequence encoded by the four nucleotides (A, T, C, and G) and shares similarity with McShea’s^18^ operational definition of biological complexity, or the degree to which the parts of a morphological structure differ from each other. *SCC*_*SW*_ may account for the complexity due to the partition of the genome into GC-rich and GC-poor segments (e.g., the isochores), which are known to be associated with many functionally relevant properties such as gene density, gene length, retrotransposon density, or recombination frequency^41–46^. Thus, *SCC*_*SW*_ might capture the genome information gained throughout evolution by the selective forces acting on these important functional elements. On the other hand, *SCC*_*RY*_ accounts for the complexity due to the partition of the genome into segments of different purine/pyrimidine richness. Such strand asymmetries are less directly related to biological function, but this alphabet has been useful to uncover long-range correlations and analyze the evolution of fractal structure in the genomes^47–49^. Recently, a connection has been found between strand symmetry and the repetitive action of transposable elements during evolution^37^ (see also Koonin^50^ and his concept of ‘fuzzy meaning’ of sequences). The partition given by *SCC*_*KM*_ has not been associated with any biological function. Finally, *GS* and *BB* explore the maximum deviation for a given *k*-*mer* between a real and a random genome. *GS* directly compares the observed distribution of *k*-*mer* classes of a real genome with respect to that corresponding to a random one. On the other hand, by calculating the entropy differences between both groups, *BB* measures the relative entropic and anti-entropic fraction of a real genome^19^.

From a population genetics perspective, cyanobacteria can be considered proto-typical bacterial species whose populations are evolving under high effective population sizes^51^, with intermediate mutation rates between those of RNA viruses (higher mutation rate) and lower or higher eukaryotes (lower mutation rates)^52^. Therefore, natural selection is expected to play a major role in the evolution of these organisms. Irrespective of whether mutations (or any source of genetic novelty) are deleterious or beneficial, their destiny will be dictated by the deterministic action of purifying or positive selection, respectively^53,54^. This observation is highly pertinent when it comes to appropriately interpreting the phylogenetic signals observed in the metrics of complexity measures and genome parameters following the in silico evolutionary processes described by Revell et al.^55^. Considering, thus, that selection is a key force in the evolution of Cyanobacteria, most of the *K-*values estimated for the metrics may reflect the action of purifying or stabilizing selection, particularly those that are below 1 (all metrics and parameters, except *GS* and %GC). *K* from *GS* is 1, which could be interpreted either as a random drift effect or, more convincingly for this type of organism, as fluctuating selection for a relatively high rate of movement of the optimum^55^. Finally, *K* associated with %GC is much higher than one, which can also be interpreted as the result of an evolutionary process with heterogeneous peak shifts.

Importantly, our study of the evolutionary trends in Cyanobacteria by means of ridge regression found clear differences between metrics of complexity and genome parameters. Four metrics (*SCC*,* SCC*_*RY*_,* SCC*_*SW*_, and *GS*) indicate changes toward higher complexity in more evolved clades (long-branch distance with respect to the root of the tree), while *SCC*_*KM*_ does not show any signs of a trend and *BB* shows a negative trend. However, the genome parameters show no evidence of any trend (Fig. 2). These results are reinforced when comparatively analyzing trends between metrics and parameters at a lower phylogenetic resolution (i.e. in monophyletic subclades, Tables S2 and S3 and Fig. S3). Although metrics used in this work capture different aspects of the evolution of genome sequence complexity in Cyanobacteria (positive trends in *SCC*,* SCC*_*RY*_, and *GS* versus negative trends in *SCC*_*SW*_ and *SCC*_*KM*_), the genome parameters never present any positive trends (Fig. S2 and Table S2). In that respect, although some metrics capture increasing sequence complexity, genome parameters do not.

It is worth noticing that the metrics to measure sequence complexity and the associated positive driven trends have captured something different from functional comparative genomics in Cyanobacteria. One interesting case is the comparison between those Cyanobacteria species that are multicellular and develop heterocysts or akinete from those that do not develop such traits. We tested this by considering which of the species chosen in our data set have heterocyst versus non-heterocyst and akinete versus non-akinete (Table S1). The presence of heterocysts or akinete could be taken as evidence of higher complexity against its absence. We carried out a test for each one of the metrics and genome parameters to see if there were a statistically significant difference and higher value of the groups of heterocyst or akinete with respect to the groups of non-heterocyst or non-akinete, respectively (Table S1). No statistically significant difference were found for any metric (except for *SCC*_*KM*_ between akinete vs non-akinete, Mann–Withney test, *P* < 0.05). However, when comparing the average values corresponding to genome parameters (genome size, gene number and %GC), we repeatedly observed that species with heterocyst or akinete showed a statistically significant higher genome size, higher gene number, and lower %GC (Mann–Withney test, *P* < 0.05). From a functional point of view, the standard genome parameters have been found to differentiate between multicellular cyanobacteria, which is not the case for the metrics, particularly among those showing a consistent positive driven trend. (i.e., *SCC*, *GS*). These metrics are capturing something different in the genomic sequence. Take, for instance, the three species (see Fig. 4) that present the highest *SCC* values: *Cyanobacterium stanieri*, *C. aponirium*, and *Trichodesmium erythraeum.* They present a larger distance from the root even more than the SynPro clade (see Fig. 1). None of these three species, nor all the Synpro clade, have heterocysts or akinete, and all appear to present a larger distance from the root than those species harboring these traits. It is clear, then, that the positive trend we have detected is reflecting something different. We speculate that the species showing a larger distance from the root may be more evolvable than those that present a shorter distance to it.

It is interesting, on the other hand, to point out the process of selection and genome streamlining of *Synechococcus* and *Prochlorococcus* in clade 97 (SynPro clade), giving rise to more evolved shorter genomes, which are AT-rich and show a lower number of genes than the rest of Cyanobacteria (Table S1). As it can be observed, there are statistically significant negative trends in the three genome parameters but also positive trends of *SCC* (Fig. 4) and *SCC*_*RY*_ metrics (Fig. S2 and Table S2). Therefore, genome reduction in this clade does not imply loss of genome complexity; on the contrary, our study shows that this clade also has a highly complex genome sequence^56^. On the other hand, it is interesting to consider the comparison between this specialist clade with others that are generalistic, like *Microcystis *sp. (Figs. 1, 4). The genus *Microcystis* appears to be older than the Synpro clade. Both, however, have no heterocysts nor akinete (as examples of complex functionality; i.e., multicellularity) but, in general, show a higher *SCC* or *GS* metric than the multicellulars. The higher *SCC* values that we observed in the SynPro clade indicate a higher intra-genome compositional diversity in these species (i.e., a higher number of compositional segments and/or higher compositional differences among them). In the same way that a high rate of genetic variability promotes a higher evolvability^57^, it can also be considered that both groups have also developed a higher capacity to evolve, captured by some of the metrics that we have studied. On the other hand, apparently genome reduction and specialization in the SynPro clade, as already stated, is not equivalent to the loss of genome sequence complexity.

In summary, considering that selection is a major driver in the evolution of Cyanobacteria, the observed positive trends towards increasing sequence complexity captured by the *SCC*,* SCC*_*RY*_, and *GS* metrics cannot be explained, contrary to what Gould^2^ holds as a passive tendency to increase. The three tests carried out in order to demonstrate whether positive trends are passive or driven show us that the positive trend is driven and is likely due to the action of natural selection, something that we have not tested for directly. Several of the metrics gathered in this study confirm this trend in the case of the evolutionary history of Cyanobacteria.

## Methods

### Phylogenetic analysis

Ninety-one complete and nearly complete cyanobacterial genomes were downloaded from GenBank and annotated using Prokka^58^ (Table S1). To infer a phylogenomic tree we proceeded first to identify the set of homologous gene families conserved among Cyanobacteria (core genome) using get_homologues.pl pipeline^59^. For this, we used BDBH and OMCL methodologies within get_homologues.pl with the following parameters: a threshold e-value ≤ 10—^10^ for BLAST searches; a minimum percent amino acid identity > 30% between query and subject sequences; and for OMCL, we set the inflation parameter (I) set to 2.0. The consensus core-genome was inferred by the intersection of BDBH and OMCL gene families. To select high-quality phylogenetic markers from the core-genome (i.e. those gene families not showing recombination and/or horizontal gene transfer), we used the software package get_phylomarkers^60^. By this procedure, we obtained an alignment of 96 top markers comprising 36,760 amino acids. Clustal-Omega was used to align the protein sequences^61^. The multiple alignment was cured by eliminating uninformative sites and misaligned positions with Gblocks^62^. Finally, a maximum likelihood phylogeny was reconstructed using PhyML^63^ with LG model + I (estimation of invariant sites) + G (gamma distribution) as selected by ProtTest3^64^. The root was located on the branch connecting both *Gloebacter* spp. to the rest of the cyanobacteria. This location of the root is based on cytologic (for instance, *Gloebacter *spp. lacks thylakoids) as well phylogenetic and molecular clock analyses^32–34,65^.

### Genome sequence complexity metrics

#### SCC

Sequence Compositional Complexity of genomes was calculated by using a two-step process. We first obtained the non-overlapping compositional domains comprising the genome sequence, and then applied an entropic complexity measurement able to account for the heterogeneity of such compositional domains. The compositional domains of a given genome sequence are obtained through a segmentation algorithm that was properly designed^66^ by using the Jensen-Shannon entropic divergence^67,68^ to split the sequence—and iteratively the sub-sequences- into non-overlapping compositional domains which, at a given statistical significance, *s*, are homogeneous and compositionally different from the neighboring domains. It is worth mentioning that the segmentation algorithm we used, and hence the *SCC* complexity values derived from it, are invariable to sequence orientation, as Shannon entropy is invariant under symbol interchange.

Note also that the statistical significance level *s*, is the probability that the difference between each pair of adjacent domains is not due to statistical fluctuations. By changing this parameter one can obtain the underlying distribution of segment lengths and nucleotide compositions at different levels of detail^69^ thus fulfilling one of the key requirements for complexity measures^14^. Improvements to this segmentation algorithm also allow to segment long-range correlated sequences^70^. Full details of the segmentation algorithm have been published elsewhere^71,72^. Implementation details, as well as source codes and executable binaries for different operating systems can be downloaded from: https://github.com/bioinfoUGR/segment and https://github.com/bioinfoUGR/isofinder.

Once a genome sequence was segmented into *n* compositional domains, we computed *SCC* as:

$$ SCC = H\left( S \right) - \mathop \sum \limits_{i = 1}^{n} \frac{{G_{i} }}{G}H\left( {S_{i} } \right) $$

where *S* denotes the whole genomes and *G* its length, *G*_*i*_ the length of the *i* th domain, *S*_*i*_. $H\left( \cdot \right) = - \sum flog_{2} f$ is the Shannon entropy of the distribution of relative frequencies of symbol occurrences, *f*, in the corresponding (sub) sequence^17^. It should be noted that the above expression is the same one than that used in the segmentation process, applying it to the tentative two new subsequences (*n* = 2) to be obtained in each step. Thus, the two parts of the *SCC* computation are based on the same theoretical background.

We apply the above two-step procedure to each of the entire four-symbol cyanobacterial genomes, thus obtaining a *SCC* complexity value for each of them. In addition, we also apply the same procedure to the binary sequences resulting from grouping the four nucleotides into S(C,G) versus W(A,T) or R(A,G) versus Y (T,C), or K(A,C) versus M(T,G), then obtaining *SCC*_*SW*_, *SCC*_*RY*_ and *SCC*_*KM*_ metrics, respectively. These three additional metrics are partial complexities that provide complementary views of genome complexity to that obtained with the four-symbol sequence^71,72^.

We provided additional details on the segmentation carried out in Cyanobacteria by using the UCSC Genome Browser. Genome maps of the compositional segments obtained for each Cyanobacteria genome analyzed in this paper can be found at the following link: https://sites.google.com/go.ugr.es/oliver/databases/dna-compositional-segments/cyanobacteria?authuser=0. Note that, once at UCSC Genome Browser, the user can obtain a complete list of segment coordinates for each genome in plain text by clicking on Tools: Table Browser.

*BB.* Biobit is an informative measure of the complexity of a genome, which is a generalized logistic map that balances the entropic and anti-entropic components of genomes and appears to be related to their evolutionary dynamics. *BB* compares genomes of size *n* with random genomes of the same size to establish a measure of its complexity. More precisely, *BB* is a metric of genome sequence complexity that is derived from the comparison between the *k*-*mer* that yields the maximum entropy of a given random genome and the corresponding entropy of the real genome of the same length^19^. The authors demonstrated that the entropy of a real genome of length *G, E*_2*L*(*G*)_ takes a value between the maximum (2*log*_4_(*G*) or 2*L*(*G*)) and the minimum (*L*(*G*)) entropy. On the other hand, the authors define and measure two additional components, that they call entropic (*E*(*G*)) and anti-entropic (*A*(*G*)) of a real genome, in such a way that *A*(*G*) + *E*(*G*) = *L*(*G*). Then, the entropy of those components are given by *E*(*G*) = *E*_2*L*(*G*)_ − 2*L*(*G*) and *A*(*G*) = 2*L*(*G*) − *E*_2*L*(*G*)_, respectively. The *BB* of a genome (*BB*(*G*)) is a non-linear combination of the two entropic and anti-entropic components given by:

$$ BB\left( G \right) = \sqrt[{}]{L\left( G \right)}\sqrt[{}]{{\frac{A\left( G \right)}{{L\left( G \right)}}}}\left( {1 - 2\frac{A\left( G \right)}{{L\left( G \right)}}} \right)^{3} , $$

where $\frac{A\left( G \right)}{{L\left( G \right)}}$ is the anti-entropic fraction of the genome and $1 - 2\frac{A\left( G \right)}{{L\left( G \right)}}$ is the corresponding entropic fraction. Both components vary between 0 and 1. Implementation details, as well as source codes, can be downloaded from https://www.uv.es/~varnau/adn/Biobit32B.c.

#### GS

The Chaos Game Representation (*CGR*)^21,22^ is an image derived from a genome where each point of the image corresponds to a given *k*-*mer* level of analysis. If the genome sequence is a random collection of bases, the *CGR* will be a uniformly filled square image. On the bases of building a *CGR* for a particular genome, we define a corresponding Genomic Signature (*GS*) that is a numerical value obtained for a particular *k*-*mer* level by comparing point-by-point the difference between the *CGR*’s of a real genome and a random genome of the same length. In order to make it comparable, the pixel values of the images are normalized. As stated, the size of the images generated depends on the *k*-*mer* used. For a given *k*-*mer*, we have 4^*k*^ different words and the corresponding image 4^*k*^ pixels too. To build a frequency table for each *k*-*mer* minus the expected frequency for a random genome is equivalent to the difference between the *CGR* images of a real and a random genome. In fact, if *G* is the size of the genome to analyze, the expected value (*EV*) for a given *k*-*mer* is given by *EV* = (*G-k* + 1)/(4^*k*^). This value is used to normalize to 1 the values of the *k*-*mers* obtained for each of the genomes analyzed. We then define the *GS* as*:*

$$ GS = max_{k} \mathop \sum \limits_{{{\text{i}} = 1}}^{{4^{k} }} \left| {\frac{{P_{i} }}{EV} - 1} \right| $$

where *P*_*i*_ is the relative frequency of the *k*-*mer i*. Implementation details, as well as source codes, can be downloaded from https://www.uv.es/~varnau/adn/word_chaos_GS.c.

#### Standard genome parameters

Finally, we have also included three standard genome parameters: genome size, %*GC* and number of genes.

### Phylogenetic signal

We used the phylogenetic tree of Cyanobacteria to test the existence of a phylogenetic signal in the genome complexity metrics and genome parameters through Blomberg et al*.*^73 ^K-statistic in the picante package for R^74^. *K* ranges from 0 to ∞. *K* values significantly higher than zero are indicative of the presence of a phylogenetic signal or, in other words, that closely related species resemble more in the studied trait than expected by chance. *K* = 1 is the value expected under Brownian evolution.

### Phylogenetic correlations

We have examined the correlation between genome parameters and metrics of genome complexity after correcting the phylogenetic signal. Pearson *r* value between variables was computed as the phylogenetic trait variance–covariance matrix between two variables and significance tested against a *t*-distribution with *n* − 2 degrees of freedom. We used the R code provided by Liam Revell to perform Pearson correlation with phylogenetic data (https://blog.phytools.org/2017/08/pearson-correlation-with-phylogenetic.html). The *P* value obtained with this procedure is the same as that provided by a phylogenetic generalized linear square model. As we run multiple phylogenetic correlations, we corrected *P* values by false discovery rates.

### Evolutionary trends

We tested the existence of an evolutionary trend in the genomic complexity measures and genome parameters by fitting a ridge regression of each of these genomic values against tip-to-root or node-to-root distances. The *search.trend* function in the RRphylo package^75^ performs a phylogenetic ridge regression between the trait values of the tips/nodes of a phylogenetic tree and their distance to the root. The values of traits (in our case, genomic complexity and genome parameters) on internal nodes of the tree were reconstructed by the RRphylo package by applying a ridge regression for continuous ancestral character estimation, as explained in^76^. Similar to other ancestral reconstruction methods, ancestral states are calculated as a weighted average of the tip values while taking into account the phylogenetic correlation structure of the data. However, ridge regression accounts for varying rates of evolution in different regions of the tree and estimates them with ancestral characters simultaneously. The significance of the ridge regression slope was tested against 10,000 slopes obtained after simulating a simple (i.e., no-trend) Brownian evolution of the trait in our phylogenetic tree^75^.

### Continuous character mapping

We used two functions (*contMap* and *fastAnc*) from the *phytools* R package^77^. The *contMap* R function allows plotting a tree with a mapped continuous character, such as any of our complexity measures. Mapping is accomplished by estimating states at internal nodes using maximum likelihood with the function *fastAnc* and interpolating the states along each edge using Equation 2 of^78^.

### Testing trends: passive or driven

To unravel whether the positive trends are passive or driven we have applied three types of tests, called the minimum, the ancestor–descendant and the subclade test, respectively^3,36^. These tests are well known in paleontology and evolutionary biology and, to the best of our knowledge, this is the first time they have been applied to genome evolutionary analyses. To gain a better understanding of the positive trends we have also applied those tests for comparative purposes to the metrics and genome parameters that do not show evidence of such a positive evolutionary trend.

#### Minimum test

Regarding the minimum test, we have applied three types of proofs. The first one evaluates if a positive skewness of the entire phylum gives support to the existence of a left wall. It is expected that if the minimum value of a given metric or parameter delimiting the left wall increases with evolutionary time, then the trend will probably be driven. To evaluate this, we considered as the minimum the estimated value of the most basal clade, *x*_*b*_, for each metric/parameter (Fig. 1). In the second proof of the minimum test we measure |*x*_*d*_ − *x*_*b*_|, the absolute difference between descendants’ clades and the most basal clade in order to see if whether there is a statistical difference between those clades that are higher or lower than the basal clade, *x*_*b*_. Finally, the third proof of the minimum test, examines if there is a statistical difference between the average value of the absolute difference (|*x*_*d*_ − *x*_*b*_|) of a given metric or parameter higher or lower than *x*_*b*_.

#### The ancestor–descendant test

According to Gould^2^, the ancestor–descendant test is the most appropriate one to discover whether positive trends are passive or driven. McShea^36^ indicates that in a passive system, increases and decreases should be the same, whereas in a driven trend the number of increases should occur more often. To test this, we tabulated the derived clades for all possible nodes and whether they present a higher, lower, or equal value of the metric/parameter than the ancestral clade corresponding to each node. In order to avoid bias due to proximity to the putative left wall, McShea^36^ recommends applying the test only to those clades where both ancestor and descendent are higher than the average value of the metric/parameter.

#### The sub-clade test

The final test applied is the sub-clade test. According to McSchea^18^ if the parent distribution is skewed (see histograms of Fig. 3; Table 3) and the mean skew of a sub-clade drawn from the right tail is also skewed, the system is probably driven. For this test, we have applied two types of proofs. First, we tested whether the trend observed at the phylum level is also observed in four selected monophyletic clades (colored species in Fig. 1) and second, we have also applied the skewness test proposed by McShea^18^ properly to the entire phylum. Regarding the second proof for the sub-clade test, we followed the criteria given by McShea^36^ whereby the monophyletic sub-clade drawn from the right tail of the entire distribution should have a statistically significant average (median) higher value than the one corresponding to the entire phylum.

Basic statistical analyses and graphs were performed using Origin (OriginLab Corporation, Northampton, MA, USA) and R (R Core Team (2019). R: A language and environment for statistical computing. R Foundation for Statistical Computing, Vienna, Austria. URL https://www.R-project.org/).

## Supplementary information

Supplementary Information.

## Data Availability

All data generated or analysed during this study are included in this published article (and its Supplementary Information files).
