# Supplementary material for: Driven progressive evolution of genome sequence complexity in Cyanobacteria
Source: Sci Rep. 2020 Nov 4;10:19073. doi: 10.1038/s41598-020-76014-4 (PMC7643063; doi:10.1038/s41598-020-76014-4)
Supplement: Supplementary file 1 — Supplementary Information. [file 41598_2020_76014_MOESM1_ESM.pdf]

Driven progressive evolution of genome complexity in Cyanobacteria

Andrés Moya, José L. Oliver, Miguel Verdú, Luis Delaye, Vicente Arnau, Pedro Bernaola-Galván, Rebeca de la Fuente, Wladimiro Díaz, Cristina Gómez-Martín, Francisco M. González, Amparo Latorre, Ricardo Lebrón & Ramón Román-Roldán

Species	NCBIRef	Assembly accession	Heterocyst	Akinete	SCC	SCC_SW	SCC_RY	SCC_KM	GS	BB	Genome size	%GC	No. of genes
Leptolyngbya_boryana_dg5	AP014642.1	GCF_002142495.1	no	no	0.0055	0.0003	0.0003	0.0016	0.5944	0.1451	6176365	46.99	6144
Calothrix_sp._NIES_2098	AP018172.1	GCF_002368175.1	yes (by inference)	yes (by inference)	0.0094	0.0051	0.0001	0.0002	0.6425	0.1706	8656060	41.20	7082
Chondrocystis_sp_NIES_4102	AP018281.1	GCF_002368355.1	no	no	0.0111	0.0019	0.0019	0.0010	0.7459	0.2086	4516676	36.53	4198
Thermosynechococcus_elongatus_BP_1	BA000039.2	GCF_000011345.1	no	no	0.0047	0.0001	0.0001	0.0006	0.6483	0.3032	2593857	53.92	2476
Gloeobacter_violaceus_PCC_7421	BA000045.2	GCF_000011385.1	no	no	0.0095	0.0058	0.0001	0.0003	0.8216	0.1380	4659019	62.00	4430
Prochlorococcus_marinus_subsp._pastoris_str._CCMP1986	BX548174.1	GCF_000011465.1	no	no	0.0173	0.0049	0.0082	0.0006	0.8197	0.0726	1657990	30.80	1790
Anabaena_sp._90_Chromosome_chANA01	CP003284.1	GCF_000312705.1	yes	yes (by inference)	0.0149	0.0049	0.0020	0.0003	0.7406	0.3151	4329264	38.10	4531
Rivularia_sp._PCC_7116	CP003549.1	GCF_000316665.1	yes	no	0.0128	0.0026	0.0015	0.0011	0.7498	0.1990	8698463	37.54	6526
Pseudanabaena_sp._PCC_7367	CP003592.1	GCF_000317065.1	no	no	0.0079	0.0007	0.0002	0.0011	0.6684	0.1972	4557046	46.31	3877
Oscillatoria_nigro_viridis_PCC_7112	CP003614.1	GCF_000317475.1	no	no	0.0163	0.0045	0.0009	0.0043	0.5948	0.3362	7479014	45.87	6408
Gloeocapsa_sp._PCC_7428	CP003646.1	GCF_000317555.1	no	no	0.0085	0.0034	0.0000	0.0008	0.5904	0.1100	5431448	43.27	4996
Cyanobacterium_stanieri_PCC_7202	CP003940.1	GCF_000317655.1	no	no	0.0257	0.0072	0.0008	0.0067	0.7436	0.1744	3163381	38.66	2837
Leptolyngbya_sp._PCC_7376	CP003946.1	GCF_000316605.1	no	no	0.0078	0.0023	0.0003	0.0014	0.6078	0.3789	5125950	43.87	4167
Microcystis_panniformis_FACHB_1757	CP011339.1	GCF_001264245.1	no	no	0.0173	0.0067	0.0021	0.0006	0.6771	0.5209	5686839	42.35	5974
Arthrospira_sp_PCC_8005	FO818640.1	GCF_000973065.1	no	no	0.0136	0.0040	0.0027	0.0023	0.6321	0.5083	6228153	44.73	5293
Synechocystis_sp._PCC_6803	NC_000911.1	GCF_001318385.1	no	no	0.0109	0.0060	0.0005	0.0003	0.6692	0.1887	3573470	47.72	3204
Nostoc_sp._PCC_7120	NC_003272.1	GCF_000009705.1	yes	yes	0.0107	0.0043	0.0003	0.0001	0.6197	0.2037	6413771	41.35	5842
Prochlorococcus_marinus_subsp_marinus_str_CCMP1375	NC_005042.1	GCF_000007925.1	no	no	0.0116	0.0028	0.0043	0.0023	0.7325	0.0679	1751080	36.44	1882
Synechococcus_sp._WH_8102	NC_005070.1	GCF_000195975.1	no	no	0.0176	0.0124	0.0006	0.0017	0.7361	0.1826	2434428	59.41	2513
Prochlorococcus_marinus_str_MIT_9313	NC_005071.1	GCF_000011485.1	no	no	0.0156	0.0077	0.0005	0.0049	0.5902	0.1334	2410873	50.74	2369
Synechococcus_elongatus_PCC_6301	NC_006576.1	GCF_000010065.1	no	no	0.0067	0.0043	0.0000	0.0003	0.6473	0.0564	2696255	55.48	2602
Prochlorococcus_marinus_str._NATL2A	NC_007335.2	GCF_000012465.1	no	no	0.0153	0.0046	0.0051	0.0018	0.7571	0.1461	1842899	35.12	1953
Trichormus_variabilis_ATCC_29413	NC_007413.1	GCF_000204075.1	yes	yes	0.0105	0.0046	0.0004	0.0004	0.6206	0.2421	6365727	41.42	5676
Synechococcus_sp._CC9902	NC_007513.1	GCF_000012505.1	no	no	0.0156	0.0092	0.0008	0.0034	0.6111	0.0690	2234828	54.16	2337
Synechococcus_sp._CC9605	NC_007516.1	GCF_000012625.1	no	no	0.0167	0.0116	0.0003	0.0015	0.7302	0.1029	2510659	59.22	2665
Prochlorococcus_marinus_str_MIT_9312	NC_007577.1	GCF_000012645.1	no	no	0.0176	0.0052	0.0084	0.0007	0.8165	0.0852	1709204	31.21	1826
Synechococcus_elongatus_PCC_7942	NC_007604.1	GCF_000012525.1	no	no	0.0067	0.0043	0.0001	0.0003	0.6474	0.0523	2695903	55.47	2685
Synechococcus_sp_JA_3_3Ab	NC_007775.1	GCF_000013205.1	no	no	0.0105	0.0040	0.0000	0.0008	0.7850	0.3043	2932766	60.24	2611
Synechococcus_sp_JA_2_3B_a_2_13	NC_007776.1	GCF_000013225.1	no	no	0.0086	0.0026	0.0001	0.0020	0.7515	0.2847	3046682	58.45	2692
Trichodesmium_erythraeum_IMS101	NC_008312.1	GCF_000014265.1	no	no	0.0228	0.0085	0.0054	0.0037	0.8707	0.4000	7750108	34.14	4549
Synechococcus_sp._CC9311	NC_008319.1	GCF_000014585.1	no	no	0.0166	0.0091	0.0008	0.0042	0.5892	0.1273	2606748	52.45	2663
Prochlorococcus_marinus_str._AS9601	NC_008816.1	GCF_000015645.1	no	no	0.0178	0.0051	0.0085	0.0007	0.8132	0.0896	1669886	31.32	1784
Prochlorococcus_marinus_str._MIT_9515	NC_008817.1	GCF_000015665.1	no	no	0.0178	0.0055	0.0084	0.0007	0.8245	0.0798	1704176	30.79	1794
Prochlorococcus_marinus_str._NATL1A	NC_008819.1	GCF_000015685.1	no	no	0.0157	0.0047	0.0052	0.0019	0.7612	0.1136	1864731	34.98	1976
Synechococcus_sp._RCC307	NC_009482.1	GCF_000063525.1	no	no	0.0100	0.0053	0.0002	0.0020	0.7824	0.0620	2224914	60.84	2388
Acaryochloris_marina_MBIC11017	NC_009925.1	GCF_000018105.1	no	no	0.0087	0.0043	0.0004	0.0011	0.4988	0.3259	6503724	47.25	7163
Microcystis_aeruginosa_NIES_843	NC_010296.1	GCF_000010625.1	no	no	0.0184	0.0072	0.0020	0.0005	0.6789	0.5272	5842795	42.33	5190
Synechococcus_sp._PCC_7002	NC_010475.1	GCF_000019485.1	no	no	0.0105	0.0031	0.0000	0.0017	0.6137	0.0924	3008047	49.63	3148
Cyanothece_sp._ATCC_51142	NC_010546.1	GCF_000017845.1	no	no	0.0159	0.0078	0.0012	0.0012	0.7318	0.1957	4934271	37.88	4942
Nostoc_punctiforme_PCC_73102	NC_010628.1	GCF_000020025.1	yes	yes	0.0095	0.0046	0.0007	0.0000	0.6249	0.2768	8234322	41.41	6984
Cyanothece_sp._PCC_8801	NC_011726.1	GCF_000021805.1	no	no	0.0149	0.0079	0.0000	0.0004	0.6980	0.2034	4679413	39.76	4326
Cyanothece_sp._PCC_7424	NC_011729.1	GCF_000021825.1	no	no	0.0148	0.0045	0.0015	0.0016	0.7157	0.2530	5942652	38.61	5603
Cyanothece_sp._PCC_7425	NC_011884.1	GCF_000022045.1	no	no	0.0086	0.0049	0.0001	0.0002	0.6005	0.2504	5374574	50.79	5202
Cyanothece_sp._PCC_8802	NC_013161.1	GCF_000024045.1	no	no	0.0146	0.0078	0.0000	0.0002	0.6958	0.1516	4669813	39.82	4371
Candidatus_Atelocyanobacterium_thalassa_isolate_ALOHA	NC_013771.1	GCF_000025125.1	no	no	0.0106	0.0025	0.0014	0.0000	0.7835	0.0141	1443806	31.12	1148
Nostoc_azollae_0708	NC_014248.1	GCF_000196515.1	yes	yes	0.0123	0.0016	0.0018	0.0004	0.7431	0.4708	5354700	38.45	3669
Cyanothece_sp._PCC_7822	NC_014501.1	GCF_000147335.1	no	no	0.0131	0.0024	0.0005	0.0019	0.6672	0.2010	6091620	40.22	6683
Arthrospira_platensis_NIES_39	NC_016640.1	GCF_000210375.1	no	no	0.0150	0.0044	0.0035	0.0038	0.6427	0.5361	6788435	43.65	5872
Cyanobium_gracile_PCC_6307	NC_019675.1	GCF_000316515.1	no	no	0.0161	0.0114	0.0009	0.0005	0.9722	0.1545	3342364	68.71	3191
Nostoc_sp._PCC_7107	NC_019676.1	GCF_000316625.1	yes	yes	0.0095	0.0038	0.0002	0.0006	0.6642	0.2174	6329823	40.36	5192
Synechococcus_sp_PCC_6312	NC_019680.1	GCF_000316685.1	no	no	0.0107	0.0046	0.0002	0.0018	0.6020	0.1331	3697276	48.52	3528
Calothrix_sp._PCC_7507	NC_019682.1	GCF_000316575.1	yes	yes	0.0094	0.0042	0.0003	0.0001	0.6144	0.2470	7023215	42.25	5836
Nostoc_sp._PCC_7524	NC_019684.1	GCF_000316645.1	yes	yes	0.0113	0.0047	0.0008	0.0010	0.6332	0.2669	6635030	41.53	5405
Pleurocapsa_sp._PCC_7327	NC_019689.1	GCF_000317025.1	no	no	0.0118	0.0061	0.0005	0.0007	0.6080	0.2026	4986817	45.19	4271
Oscillatoria_acuminata_PCC_6304	NC_019693.1	GCF_000317105.1	no	no	0.0128	0.0038	0.0011	0.0018	0.5971	0.2879	7689443	47.60	5879
Chroococcidiopsis_thermalis_PCC_7203	NC_019695.1	GCF_000317125.1	no	no	0.0100	0.0042	0.0006	0.0005	0.5662	0.1985	6315792	44.44	5716
Chamaesiphon_minutus_PCC_6605	NC_019697.1	GCF_000317145.1	no	no	0.0095	0.0037	0.0003	0.0005	0.5962	0.3231	6284095	45.73	6013
Synechococcus_sp._PCC_7502	NC_019702.1	GCF_000317085.1	no	no	0.0058	0.0012	0.0000	0.0010	0.6733	0.3091	3510253	40.62	3442
Geitlerinema_sp._PCC_7407	NC_019703.1	GCF_000317045.1	no	no	0.0187	0.0118	0.0003	0.0012	0.7190	0.1261	4681111	58.46	3789
Microcoleus_sp._PCC_7113	NC_019738.1	GCF_000317515.1	no	no	0.0092	0.0034	0.0003	0.0004	0.4995	0.2215	7470429	46.21	6350
Stanieria_cyanosphaera_PCC_7437	NC_019748.1	GCF_000317575.1	no	no	0.0092	0.0032	0.0002	0.0006	0.7960	0.1622	5041209	35.95	4758
Calothrix_sp._PCC_6303	NC_019751.1	GCF_000317435.1	yes	no	0.0123	0.0036	0.0017	0.0012	0.6702	0.2512	6767834	39.80	5465
Crinalium_epipsammum_PCC_9333	NC_019753.1	GCF_000317495.1	no	no	0.0099	0.0026	0.0014	0.0016	0.6482	0.2338	5315554	40.16	4728
Cylindrospermum_stagnale_PCC_7417	NC_019757.1	GCF_000317535.1	yes	yes	0.0118	0.0053	0.0009	0.0004	0.6193	0.2233	7003560	42.30	6158
Anabaena_cylindrica_PCC_7122_c	NC_019771.1	GCF_000317695.1	yes	yes	0.0127	0.0049	0.0014	0.0012	0.7081	0.2648	6395836	38.80	5834
Cyanobacterium_aponinum_PCC_10605	NC_019776.1	GCF_000317675.1	no	no	0.0216	0.0061	0.0031	0.0002	0.8160	0.2883	4114099	34.96	3426
Halothece_sp._PCC_7418	NC_019779.1	GCF_000317635.1	no	no	0.0102	0.0030	0.0009	0.0013	0.6501	0.2452	4179170	42.92	3710
Dactylococcopsis_salina_PCC_8305	NC_019780.1	GCF_000317615.1	no	no	0.0165	0.0035	0.0043	0.0018	0.6906	0.3888	3781008	42.44	3429
Gloeobacter_kilaueensis_JS1	NC_022600.1	GCF_000484535.1	no	no	0.0116	0.0067	0.0002	0.0002	0.8134	0.1802	4724791	60.54	4336
Thermosynechococcus_sp._NK55a	NC_023033.1	GCF_000505665.1	no	no	0.0045	0.0014	0.0001	0.0004	0.6403	0.0708	2520064	53.81	2287
Geminocystis_sp._NIES_3708	NZ_AP014815.1	GCF_001548095.1	no	no	0.0159	0.0066	0.0023	0.0000	0.8533	0.1995	3883409	32.28	3376
Geminocystis_sp._NIES_3709	NZ_AP014821.1	GCF_001548115.1	no	no	0.0174	0.0071	0.0043	0.0005	0.8448	0.3198	4150181	33.34	3587
Fischerella_sp_NIES_3754	NZ_AP017305.1	GCF_001548455.1	yes	yes	0.0127	0.0037	0.0004	0.0006	0.6563	0.1869	5821603	40.99	4584
Leptolyngbya_sp._O_77	NZ_AP017367.1	GCF_001548395.1	no	no	0.0143	0.0061	0.0002	0.0008	0.6679	0.2720	5480261	55.93	4291
Calothrix_sp._NIES_2100	NZ_AP018178.1	GCF_002368195.1	yes (by inference)	yes (by inference)	0.0105	0.0043	0.0013	0.0004	0.6439	0.2308	9909373	41.41	7574
Calothrix_sp._NIES_3974	NZ_AP018254.1	GCF_002368395.1	yes (by inference)	no (by inference)	0.0138	0.0030	0.0006	0.0025	0.6759	0.2685	5985875	41.53	4529
Calothrix_sp._NIES_4071	NZ_AP018255.1	GCF_002368455.1	yes (by inference)	no (by inference)	0.0100	0.0022	0.0011	0.0010	0.6799	0.1937	11064963	39.05	9858
Aulosira_laxa_NIES_50	NZ_AP018307.1	GCF_002368055.1	yes (by inference)	yes (by inference)	0.0105	0.0045	0.0009	0.0005	0.6713	0.2562	8460156	40.68	7124
Synechococcus_sp._WH_8109	NZ_CP006882.1	GCF_000161795.2	no	no	0.0124	0.0081	0.0003	0.0015	0.7262	0.1098	2111515	60.09	2232
Nodularia_spumigena_CCY9414	NZ_CP007203.1	GCF_000340565.2	yes	yes	0.0126	0.0043	0.0018	0.0023	0.6469	0.3625	5462271	41.19	4566
Calothrix_sp._336_3	NZ_CP011382.1	GCF_000734895.2	yes	no (by inference)	0.0114	0.0040	0.0006	0.0012	0.6534	0.2009	6283267	41.04	5232
Anabaena_sp._WA102	NZ_CP011456.1	GCF_001277295.1	yes	yes (by inference)	0.0173	0.0057	0.0023	0.0018	0.7350	0.3991	5705437	38.39	4927
Synechococcus_sp._WH_8020	NZ_CP011941.1	GCF_001040845.1	no	no	0.0162	0.0088	0.0009	0.0042	0.6027	0.0675	2661166	53.12	2725
Nostoc_piscinale_CENA21	NZ_CP012036.1	GCF_001298445.1	yes	yes (by inference)	0.0112	0.0041	0.0007	0.0012	0.6662	0.1581	7094556	40.54	5232
Microcystis_aeruginosa_NIES_2481	NZ_CP012375.1	GCF_001704955.2	no	no	0.0146	0.0065	0.0001	0.0012	0.6534	0.3254	4293006	42.91	3966
Arthrospira_platensis_YZ	NZ_CP013008.1	GCF_001611905.1	no	no	0.0146	0.0043	0.0034	0.0037	0.6396	0.5223	6520772	44.19	5551
Moorea_producens_PAL_8_15_08_1	NZ_CP017599.1	GCF_001767235.1	no	no	0.0132	0.0025	0.0023	0.0009	0.5947	0.3972	9673108	43.52	6792
Moorea_producens_JHB	NZ_CP017708.1	GCF_001854205.1	no	no	0.0136	0.0040	0.0030	0.0022	0.5959	0.3742	9373345	43.55	6690
Synechococcus_lividus_PCC_6715	NZ_CP018092.1	GCF_002754935.1	no	no	0.0049	0.0018	0.0000	0.0000	0.6114	0.2759	2659739	53.51	2227
Prochlorococcus_sp_RS01	NZ_CP018345.1	GCF_001989435.1	no	no	0.0177	0.0049	0.0085	0.0006	0.8123	0.0646	1657699	31.38	1793
Synechococcus_sp._WH_8103	NZ_LN847356.1	GCF_001182765.1	no	no	0.0172	0.0120	0.0007	0.0015	0.7394	0.2345	2429688	59.47	2492

Table S1. General genome features, genome parameters, and metrics of genome sequence complexity of Cyanobacteria genomes. "By inference" means that for those species in our dataset from which it is unknown if they have heterocyst or akinete, there is direct evidence that closely related phylogenetic species have it.

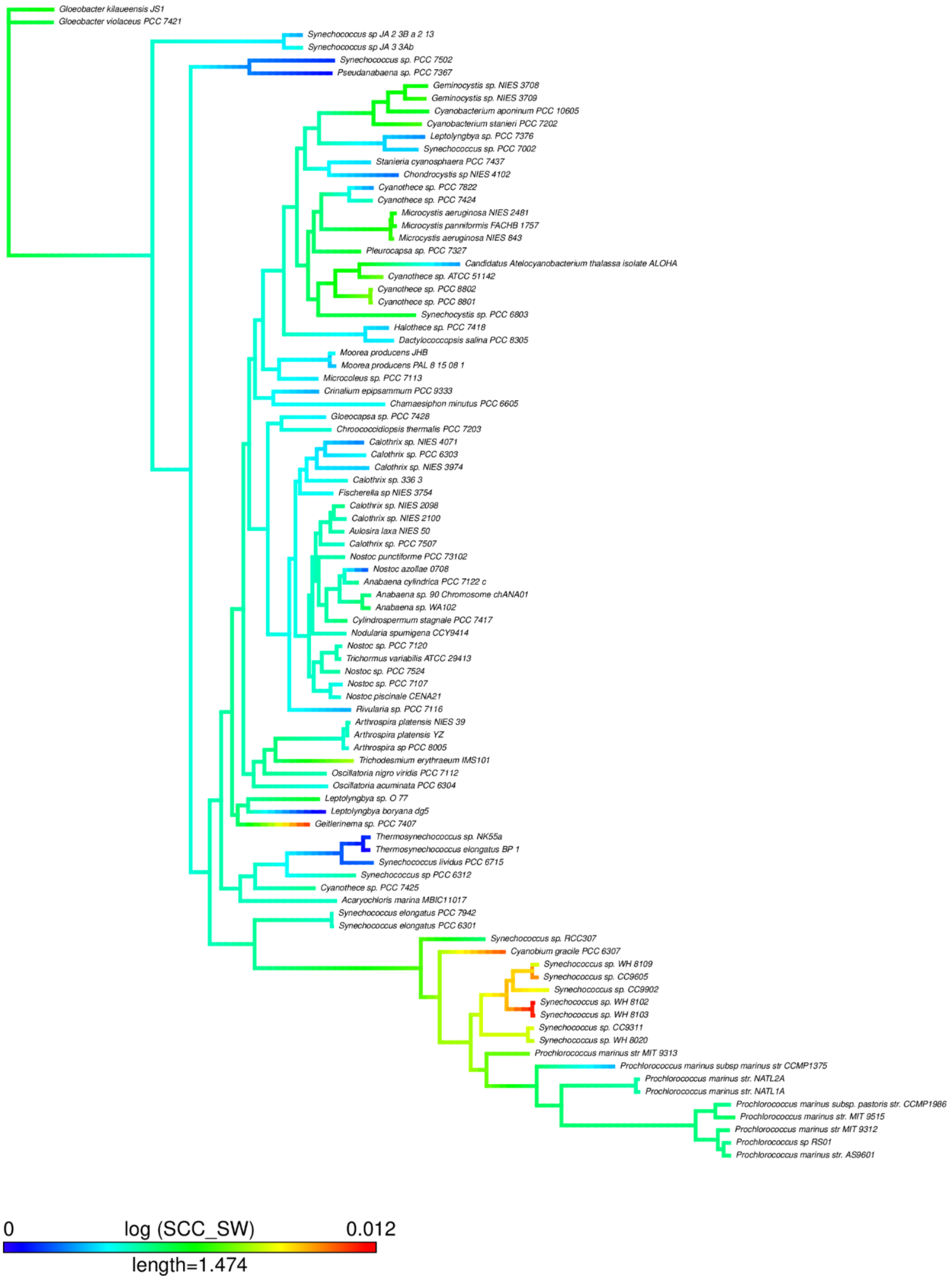
Gloeobacter kilaueensis JS1
Gloeobacter violaceus PCC 7421
Synechococcus sp JA 2 3B a 2 13
Synechococcus sp JA 3 3Ab
Synechococcus sp. PCC 7502
Pseudanabaena sp. PCC 7367
Geminocystis sp. NIES 3708
Geminocystis sp. NIES 3709
Cyanobacterium aponinum PCC 10605
Cyanobacterium stanieri PCC 7202
Leptolyngbya sp. PCC 7376
Synechococcus sp. PCC 7002
Stanieria cyanosphaera PCC 7437
Chondrocystis sp NIES 4102
Cyanothece sp. PCC 7822
Cyanothece sp. PCC 7424
Microcystis aeruginosa NIES 2481
Microcystis panniformis FACHB 1757
Microcystis aeruginosa NIES 843
Pleurocapsa sp. PCC 7327
Candidatus Atelocyanobacterium thalassa isolate ALOHA
Cyanothece sp. ATCC 51142
Cyanothece sp. PCC 8802
Cyanothece sp. PCC 8801
Synechocystis sp. PCC 6803
Halothece sp. PCC 7418
Dactylococcopsis salina PCC 8305
Moorea producens JHB
Moorea producens PAL 8 15 08 1
Microcoleus sp. PCC 7113
Crinalium epipsammum PCC 9333
Chamaesiphon minutus PCC 6605
Gloeocapsa sp. PCC 7428
Chroococcidiopsis thermalis PCC 7203
Calothrix sp. NIES 4071
Calothrix sp. PCC 6303
Calothrix sp. NIES 3974
Calothrix sp. 336 3
Fischerella sp NIES 3754
Calothrix sp. NIES 2098
Calothrix sp. NIES 2100
Aulosira laxa NIES 50
Calothrix sp. PCC 7507
Nostoc punctiforme PCC 73102
Nostoc azollae 0708
Anabaena cylindrica PCC 7122 c
Anabaena sp. 90 Chromosome chANA01
Anabaena sp. WA102
Cylindrospermum stagnale PCC 7417
Nodularia spumigena CCY9414
Nostoc sp. PCC 7120
Trichormus variabilis ATCC 29413
Nostoc sp. PCC 7524
Nostoc sp. PCC 7107
Nostoc piscinale CENA21
Rivularia sp. PCC 7116
Arthrospira platensis NIES 39
Arthrospira platensis YZ
Arthrospira sp PCC 8005
Trichodesmium erythraeum IMS101
Oscillatoria nigro viridis PCC 7112
Oscillatoria acuminata PCC 6304
Leptolyngbya sp. O 77
Leptolyngbya boryana dg5
Geitlerinema sp. PCC 7407
Thermosynechococcus sp. NK55a
Thermosynechococcus elongatus BP 1
Synechococcus lividus PCC 6715
Synechococcus sp PCC 6312
Cyanothece sp. PCC 7425
Acaryochloris marina MBIC11017
Synechococcus elongatus PCC 7942
Synechococcus elongatus PCC 6301
Synechococcus sp. RCC307
Cyanobium gracile PCC 6307
Synechococcus sp. WH 8109
Synechococcus sp. CC9605
Synechococcus sp. CC9902
Synechococcus sp. WH 8102
Synechococcus sp. WH 8103
Synechococcus sp. CC9311
Synechococcus sp. WH 8020
Prochlorococcus marinus str MIT 9313
Prochlorococcus marinus subsp marinus str CCMP1375
Prochlorococcus marinus str. NATL2A
Prochlorococcus marinus str. NATL1A
Prochlorococcus marinus subsp. pastoris str. CCMP1986
Prochlorococcus marinus str. MIT 9515
Prochlorococcus marinus str MIT 9312
Prochlorococcus sp RS01
Prochlorococcus marinus str. AS9601
0
log (SCC_SW)
0.012
length=1.474

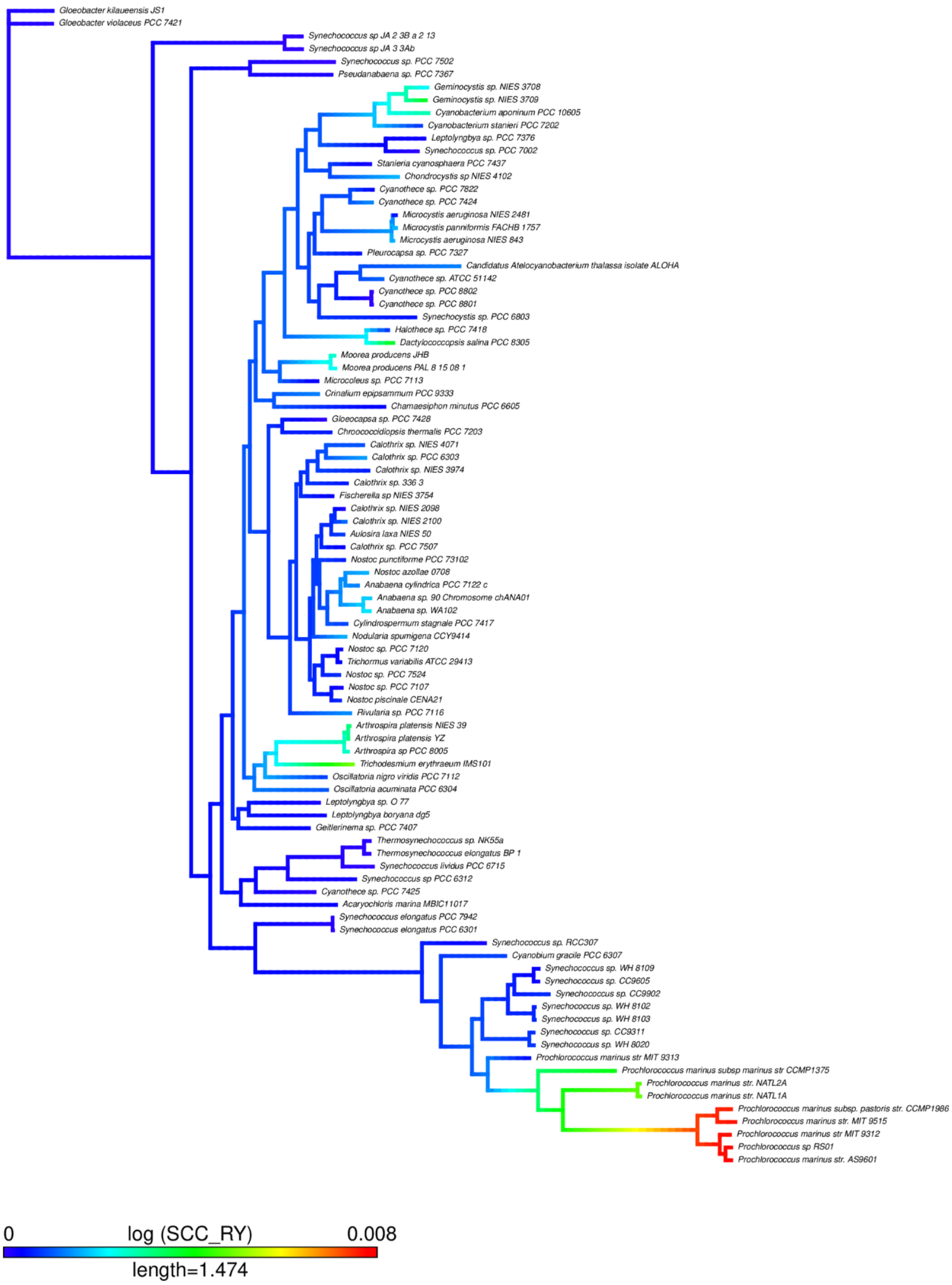
Gloeobacter kilaueensis JS1
Gloeobacter violaceus PCC 7421
Synechococcus sp JA 2 3B a 2 13
Synechococcus sp JA 3 3Ab
Synechococcus sp. PCC 7502
Pseudanabaena sp. PCC 7367
Geminocystis sp. NIES 3708
Geminocystis sp. NIES 3709
Cyanobacterium aponinum PCC 10605
Cyanobacterium stanieri PCC 7202
Leptolyngbya sp. PCC 7376
Synechococcus sp. PCC 7002
Stanieria cyanosphaera PCC 7437
Chondrocystis sp NIES 4102
Cyanothece sp. PCC 7822
Cyanothece sp. PCC 7424
Microcystis aeruginosa NIES 2481
Microcystis panniformis FACHB 1757
Microcystis aeruginosa NIES 843
Pleurocapsa sp. PCC 7327
Candidatus Atelocyanobacterium thalassa isolate ALOHA
Cyanothece sp. ATCC 51142
Cyanothece sp. PCC 8802
Cyanothece sp. PCC 8801
Synechocystis sp. PCC 6803
Halothece sp. PCC 7418
Dactylococcopsis salina PCC 8305
Moorea producens JHB
Moorea producens PAL 8 15 08 1
Microcoleus sp. PCC 7113
Crinalium epipsammum PCC 9333
Chamaesiphon minutus PCC 6605
Gloeocapsa sp. PCC 7428
Chroococcidiopsis thermalis PCC 7203
Calothrix sp. NIES 4071
Calothrix sp. PCC 6303
Calothrix sp. NIES 3974
Calothrix sp. 336 3
Fischerella sp NIES 3754
Calothrix sp. NIES 2098
Calothrix sp. NIES 2100
Aulosira laxa NIES 50
Calothrix sp. PCC 7507
Nostoc punctiforme PCC 73102
Nostoc azollae 0708
Anabaena cylindrica PCC 7122 c
Anabaena sp. 90 Chromosome chANA01
Anabaena sp. WA102
Cylindrospermum stagnale PCC 7417
Nodularia spumigena CCY9414
Nostoc sp. PCC 7120
Trichormus variabilis ATCC 29413
Nostoc sp. PCC 7524
Nostoc sp. PCC 7107
Nostoc piscinale CENA21
Rivularia sp. PCC 7116
Arthrospira platensis NIES 39
Arthrospira platensis YZ
Arthrospira sp PCC 8005
Trichodesmium erythraeum IMS101
Oscillatoria nigro viridis PCC 7112
Oscillatoria acuminata PCC 6304
Leptolyngbya sp. O 77
Leptolyngbya boryana dg5
Geitlerinema sp. PCC 7407
Thermosynechococcus sp. NK55a
Thermosynechococcus elongatus BP 1
Synechococcus lividus PCC 6715
Synechococcus sp PCC 6312
Cyanothece sp. PCC 7425
Acaryochloris marina MBIC11017
Synechococcus elongatus PCC 7942
Synechococcus elongatus PCC 6301
Synechococcus sp. RCC307
Cyanobium gracile PCC 6307
Synechococcus sp. WH 8109
Synechococcus sp. CC9605
Synechococcus sp. CC9902
Synechococcus sp. WH 8102
Synechococcus sp. WH 8103
Synechococcus sp. CC9311
Synechococcus sp. WH 8020
Prochlorococcus marinus str MIT 9313
Prochlorococcus marinus subsp marinus str CCMP1375
Prochlorococcus marinus str. NATL2A
Prochlorococcus marinus str. NATL1A
Prochlorococcus marinus subsp. pastoris str. CCMP1986
Prochlorococcus marinus str. MIT 9515
Prochlorococcus marinus str MIT 9312
Prochlorococcus sp RS01
Prochlorococcus marinus str. AS9601
0
log (SCC_RY)
0.008
length=1.474

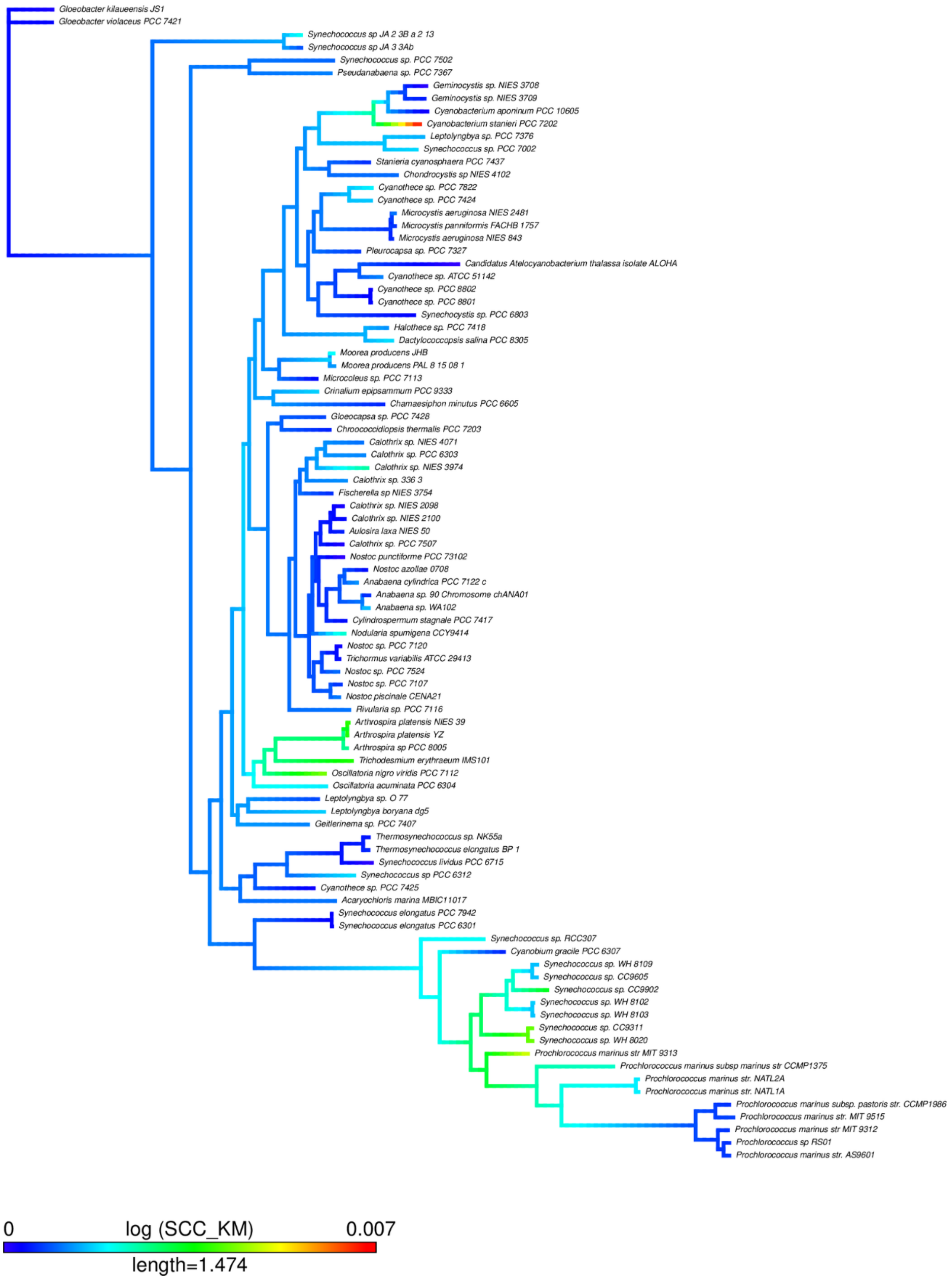
Gloeobacter kilaueensis JS1
Gloeobacter violaceus PCC 7421
Synechococcus sp JA 2 3B a 2 13
Synechococcus sp JA 3 3Ab
Synechococcus sp. PCC 7502
Pseudanabaena sp. PCC 7367
Geminocystis sp. NIES 3708
Geminocystis sp. NIES 3709
Cyanobacterium aponinum PCC 10605
Cyanobacterium stanieri PCC 7202
Leptolyngbya sp. PCC 7376
Synechococcus sp. PCC 7002
Stanieria cyanosphaera PCC 7437
Chondrocystis sp NIES 4102
Cyanothece sp. PCC 7822
Cyanothece sp. PCC 7424
Microcystis aeruginosa NIES 2481
Microcystis panniformis FACHB 1757
Microcystis aeruginosa NIES 843
Pleurocapsa sp. PCC 7327
Candidatus Atelocyanobacterium thalassa isolate ALOHA
Cyanothece sp. ATCC 51142
Cyanothece sp. PCC 8802
Cyanothece sp. PCC 8801
Synechocystis sp. PCC 6803
Halothece sp. PCC 7418
Dactylococcopsis salina PCC 8305
Moorea producens JHB
Moorea producens PAL 8 15 08 1
Microcoleus sp. PCC 7113
Crinalium epipsammum PCC 9333
Chamaesiphon minutus PCC 6605
Gloeocapsa sp. PCC 7428
Chroococcidiopsis thermalis PCC 7203
Calothrix sp. NIES 4071
Calothrix sp. PCC 6303
Calothrix sp. NIES 3974
Calothrix sp. 336 3
Fischerella sp NIES 3754
Calothrix sp. NIES 2098
Calothrix sp. NIES 2100
Aulosira laxa NIES 50
Calothrix sp. PCC 7507
Nostoc punctiforme PCC 73102
Nostoc azollae 0708
Anabaena cylindrica PCC 7122 c
Anabaena sp. 90 Chromosome chANA01
Anabaena sp. WA102
Cylindrospermum stagnale PCC 7417
Nodularia spumigena CCY9414
Nostoc sp. PCC 7120
Trichormus variabilis ATCC 29413
Nostoc sp. PCC 7524
Nostoc sp. PCC 7107
Nostoc piscinale CENA21
Rivularia sp. PCC 7116
Arthrospira platensis NIES 39
Arthrospira platensis YZ
Arthrospira sp PCC 8005
Trichodesmium erythraeum IMS101
Oscillatoria nigro viridis PCC 7112
Oscillatoria acuminata PCC 6304
Leptolyngbya sp. O 77
Leptolyngbya boryana dg5
Geitlerinema sp. PCC 7407
Thermosynechococcus sp. NK55a
Thermosynechococcus elongatus BP 1
Synechococcus lividus PCC 6715
Synechococcus sp PCC 6312
Cyanothece sp. PCC 7425
Acaryochloris marina MBIC11017
Synechococcus elongatus PCC 7942
Synechococcus elongatus PCC 6301
Synechococcus sp. RCC307
Cyanobium gracile PCC 6307
Synechococcus sp. WH 8109
Synechococcus sp. CC9605
Synechococcus sp. CC9902
Synechococcus sp. WH 8102
Synechococcus sp. WH 8103
Synechococcus sp. CC9311
Synechococcus sp. WH 8020
Prochlorococcus marinus str MIT 9313
Prochlorococcus marinus subsp marinus str CCMP1375
Prochlorococcus marinus str. NATL2A
Prochlorococcus marinus str. NATL1A
Prochlorococcus marinus subsp. pastoris str. CCMP1986
Prochlorococcus marinus str. MIT 9515
Prochlorococcus marinus str MIT 9312
Prochlorococcus sp RS01
Prochlorococcus marinus str. AS9601
0
log (SCC_KM)
0.007
length=1.474

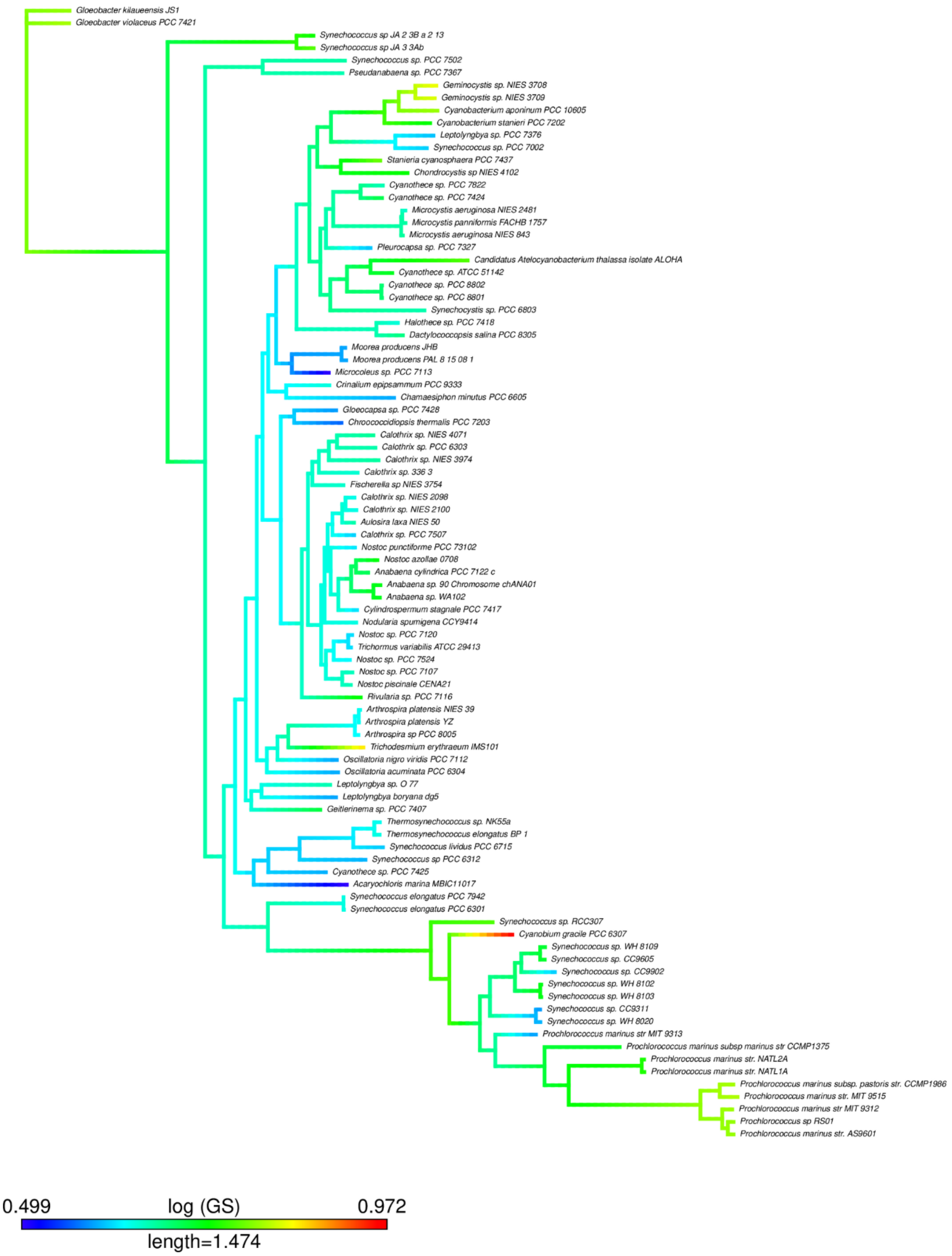

Gloeobacter kilaueensis JS1
Gloeobacter violaceus PCC 7421
Synechococcus sp JA 2 3B a 2 13
Synechococcus sp JA 3 3Ab
Synechococcus sp. PCC 7502
Pseudanabaena sp. PCC 7367
Geminocystis sp. NIES 3708
Geminocystis sp. NIES 3709
Cyanobacterium aponinum PCC 10605
Cyanobacterium stanieri PCC 7202
Leptolyngbya sp. PCC 7376
Synechococcus sp. PCC 7002
Stanieria cyanosphaera PCC 7437
Chondrocystis sp NIES 4102
Cyanothece sp. PCC 7822
Cyanothece sp. PCC 7424
Microcystis aeruginosa NIES 2481
Microcystis panniformis FACHB 1757
Microcystis aeruginosa NIES 843
Pleurocapsa sp. PCC 7327
Candidatus Atelocyanobacterium thalassa isolate ALOHA
Cyanothece sp. ATCC 51142
Cyanothece sp. PCC 8802
Cyanothece sp. PCC 8801
Synechocystis sp. PCC 6803
Halothece sp. PCC 7418
Dactylococcopsis salina PCC 8305
Moorea producens JHB
Moorea producens PAL 8 15 08 1
Microcoleus sp. PCC 7113
Crinalium epipsammum PCC 9333
Chamaesiphon minutus PCC 6605
Gloeocapsa sp. PCC 7428
Chroococcidiopsis thermalis PCC 7203
Calothrix sp. NIES 4071
Calothrix sp. PCC 6303
Calothrix sp. NIES 3974
Calothrix sp. 336 3
Fischerella sp NIES 3754
Calothrix sp. NIES 2098
Calothrix sp. NIES 2100
Aulosira laxa NIES 50
Calothrix sp. PCC 7507
Nostoc punctiforme PCC 73102
Nostoc azollae 0708
Anabaena cylindrica PCC 7122 c
Anabaena sp. 90 Chromosome chANA01
Anabaena sp. WA102
Cylindrospermum stagnale PCC 7417
Nodularia spumigena CCY9414
Nostoc sp. PCC 7120
Trichormus variabilis ATCC 29413
Nostoc sp. PCC 7524
Nostoc sp. PCC 7107
Nostoc piscinale CENA21
Rivularia sp. PCC 7116
Arthrospira platensis NIES 39
Arthrospira platensis YZ
Arthrospira sp PCC 8005
Trichodesmium erythraeum IMS101
Oscillatoria nigro viridis PCC 7112
Oscillatoria acuminata PCC 6304
Leptolyngbya sp. O 77
Leptolyngbya boryana dg5
Geitlerinema sp. PCC 7407
Thermosynechococcus sp. NK55a
Thermosynechococcus elongatus BP 1
Synechococcus lividus PCC 6715
Synechococcus sp PCC 6312
Cyanothece sp. PCC 7425
Acaryochloris marina MBIC11017
Synechococcus elongatus PCC 7942
Synechococcus elongatus PCC 6301
Synechococcus sp. RCC307
Cyanobium gracile PCC 6307
Synechococcus sp. WH 8109
Synechococcus sp. CC9605
Synechococcus sp. CC9902
Synechococcus sp. WH 8102
Synechococcus sp. WH 8103
Synechococcus sp. CC9311
Synechococcus sp. WH 8020
Prochlorococcus marinus str MIT 9313
Prochlorococcus marinus subsp marinus str CCMP1375
Prochlorococcus marinus str. NATL2A
Prochlorococcus marinus str. NATL1A
Prochlorococcus marinus subsp. pastoris str. CCMP1986
Prochlorococcus marinus str. MIT 9515
Prochlorococcus marinus str MIT 9312
Prochlorococcus sp RS01
Prochlorococcus marinus str. AS9601
0.499
log (GS)
0.972
length=1.474

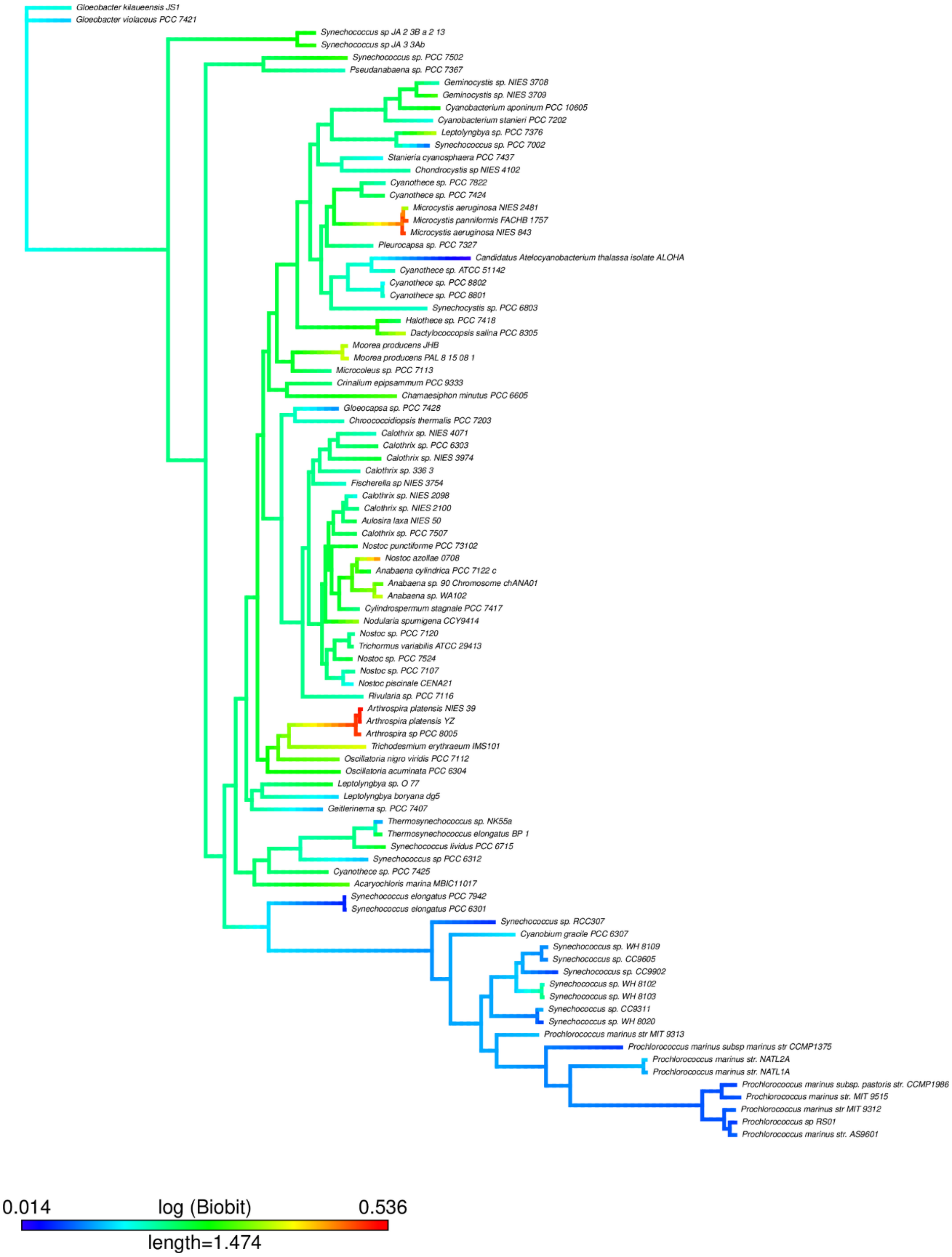
Gloeobacter kilaueensis JS1
Gloeobacter violaceus PCC 7421
Synechococcus sp JA 2 3B a 2 13
Synechococcus sp JA 3 3Ab
Synechococcus sp. PCC 7502
Pseudanabaena sp. PCC 7367
Geminocystis sp. NIES 3708
Geminocystis sp. NIES 3709
Cyanobacterium aponinum PCC 10605
Cyanobacterium stanieri PCC 7202
Leptolyngbya sp. PCC 7376
Synechococcus sp. PCC 7002
Stanieria cyanosphaera PCC 7437
Chondrocystis sp NIES 4102
Cyanothece sp. PCC 7822
Cyanothece sp. PCC 7424
Microcystis aeruginosa NIES 2481
Microcystis panniformis FACHB 1757
Microcystis aeruginosa NIES 843
Pleurocapsa sp. PCC 7327
Candidatus Atelocyanobacterium thalassa isolate ALOHA
Cyanothece sp. ATCC 51142
Cyanothece sp. PCC 8802
Cyanothece sp. PCC 8801
Synechocystis sp. PCC 6803
Halothece sp. PCC 7418
Dactylococcopsis salina PCC 8305
Moorea producens JHB
Moorea producens PAL 8 15 08 1
Microcoleus sp. PCC 7113
Crinalium epipsammum PCC 9333
Chamaesiphon minutus PCC 6605
Gloeocapsa sp. PCC 7428
Chroococcidiopsis thermalis PCC 7203
Calothrix sp. NIES 4071
Calothrix sp. PCC 6303
Calothrix sp. NIES 3974
Calothrix sp. 336 3
Fischerella sp NIES 3754
Calothrix sp. NIES 2098
Calothrix sp. NIES 2100
Aulosira laxa NIES 50
Calothrix sp. PCC 7507
Nostoc punctiforme PCC 73102
Nostoc azollae 0708
Anabaena cylindrica PCC 7122 c
Anabaena sp. 90 Chromosome chANA01
Anabaena sp. WA102
Cylindrospermum stagnale PCC 7417
Nodularia spumigena CCY9414
Nostoc sp. PCC 7120
Trichormus variabilis ATCC 29413
Nostoc sp. PCC 7524
Nostoc sp. PCC 7107
Nostoc piscinale CENA21
Rivularia sp. PCC 7116
Arthrospira platensis NIES 39
Arthrospira platensis YZ
Arthrospira sp PCC 8005
Trichodesmium erythraeum IMS101
Oscillatoria nigro viridis PCC 7112
Oscillatoria acuminata PCC 6304
Leptolyngbya sp. O 77
Leptolyngbya boryana dg5
Geitlerinema sp. PCC 7407
Thermosynechococcus sp. NK55a
Thermosynechococcus elongatus BP 1
Synechococcus lividus PCC 6715
Synechococcus sp PCC 6312
Cyanothece sp. PCC 7425
Acaryochloris marina MBIC11017
Synechococcus elongatus PCC 7942
Synechococcus elongatus PCC 6301
Synechococcus sp. RCC307
Cyanobium gracile PCC 6307
Synechococcus sp. WH 8109
Synechococcus sp. CC9605
Synechococcus sp. CC9902
Synechococcus sp. WH 8102
Synechococcus sp. WH 8103
Synechococcus sp. CC9311
Synechococcus sp. WH 8020
Prochlorococcus marinus str MIT 9313
Prochlorococcus marinus subsp marinus str CCMP1375
Prochlorococcus marinus str. NATL2A
Prochlorococcus marinus str. NATL1A
Prochlorococcus marinus subsp. pastoris str. CCMP1986
Prochlorococcus marinus str. MIT 9515
Prochlorococcus marinus str MIT 9312
Prochlorococcus sp RS01
Prochlorococcus marinus str. AS9601
0.014
log (Biobit)
0.536
length=1.474

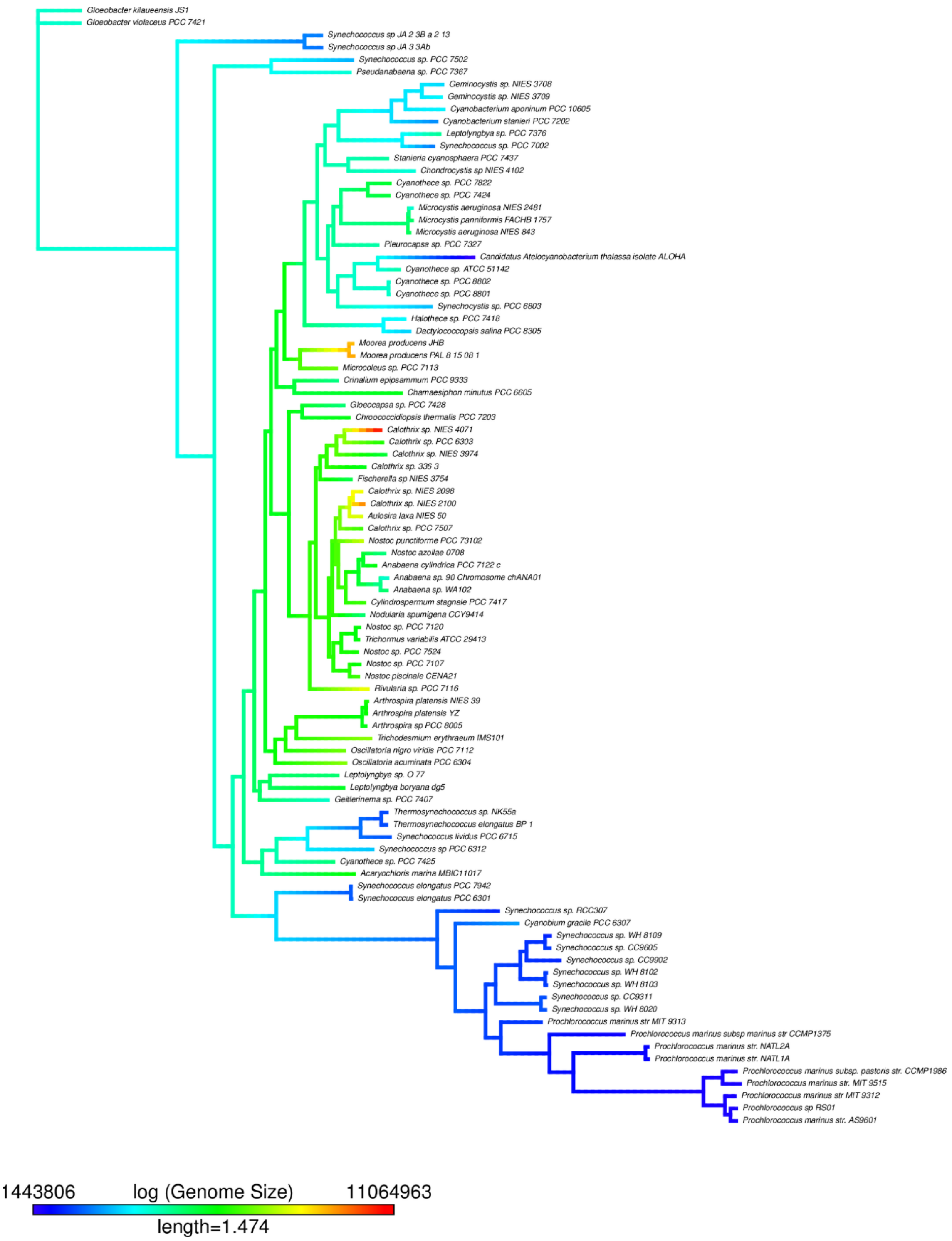

Gloeobacter kilaueensis JS1
Gloeobacter violaceus PCC 7421
Synechococcus sp JA 2 3B a 2 13
Synechococcus sp JA 3 3Ab
Synechococcus sp. PCC 7502
Pseudanabaena sp. PCC 7367
Geminocystis sp. NIES 3708
Geminocystis sp. NIES 3709
Cyanobacterium aponinum PCC 10605
Cyanobacterium stanieri PCC 7202
Leptolyngbya sp. PCC 7376
Synechococcus sp. PCC 7002
Stanieria cyanosphaera PCC 7437
Chondrocystis sp NIES 4102
Cyanothece sp. PCC 7822
Cyanothece sp. PCC 7424
Microcystis aeruginosa NIES 2481
Microcystis panniformis FACHB 1757
Microcystis aeruginosa NIES 843
Pleurocapsa sp. PCC 7327
Candidatus Atelocyanobacterium thalassa isolate ALOHA
Cyanothece sp. ATCC 51142
Cyanothece sp. PCC 8802
Cyanothece sp. PCC 8801
Synechocystis sp. PCC 6803
Halothece sp. PCC 7418
Dactylococcopsis salina PCC 8305
Moorea producens JHB
Moorea producens PAL 8 15 08 1
Microcoleus sp. PCC 7113
Crinalium epipsammum PCC 9333
Chamaesiphon minutus PCC 6605
Gloeocapsa sp. PCC 7428
Chroococcidiopsis thermalis PCC 7203
Calothrix sp. NIES 4071
Calothrix sp. PCC 6303
Calothrix sp. NIES 3974
Calothrix sp. 336 3
Fischerella sp NIES 3754
Calothrix sp. NIES 2098
Calothrix sp. NIES 2100
Aulosira laxa NIES 50
Calothrix sp. PCC 7507
Nostoc punctiforme PCC 73102
Nostoc azollae 0708
Anabaena cylindrica PCC 7122 c
Anabaena sp. 90 Chromosome chANA01
Anabaena sp. WA102
Cylindrospermum stagnale PCC 7417
Nodularia spumigena CCY9414
Nostoc sp. PCC 7120
Trichormus variabilis ATCC 29413
Nostoc sp. PCC 7524
Nostoc sp. PCC 7107
Nostoc piscinale CENA21
Rivularia sp. PCC 7116
Arthrospira platensis NIES 39
Arthrospira platensis YZ
Arthrospira sp PCC 8005
Trichodesmium erythraeum IMS101
Oscillatoria nigro viridis PCC 7112
Oscillatoria acuminata PCC 6304
Leptolyngbya sp. O 77
Leptolyngbya boryana dg5
Geitlerinema sp. PCC 7407
Thermosynechococcus sp. NK55a
Thermosynechococcus elongatus BP 1
Synechococcus lividus PCC 6715
Synechococcus sp PCC 6312
Cyanothece sp. PCC 7425
Acaryochloris marina MBIC11017
Synechococcus elongatus PCC 7942
Synechococcus elongatus PCC 6301
Synechococcus sp. RCC307
Cyanobium gracile PCC 6307
Synechococcus sp. WH 8109
Synechococcus sp. CC9605
Synechococcus sp. CC9902
Synechococcus sp. WH 8102
Synechococcus sp. WH 8103
Synechococcus sp. CC9311
Synechococcus sp. WH 8020
Prochlorococcus marinus str MIT 9313
Prochlorococcus marinus subsp marinus str CCMP1375
Prochlorococcus marinus str. NATL2A
Prochlorococcus marinus str. NATL1A
Prochlorococcus marinus subsp. pastoris str. CCMP1986
Prochlorococcus marinus str. MIT 9515
Prochlorococcus marinus str MIT 9312
Prochlorococcus sp RS01
Prochlorococcus marinus str. AS9601
1443806
log (Genome Size)
11064963
length=1.474

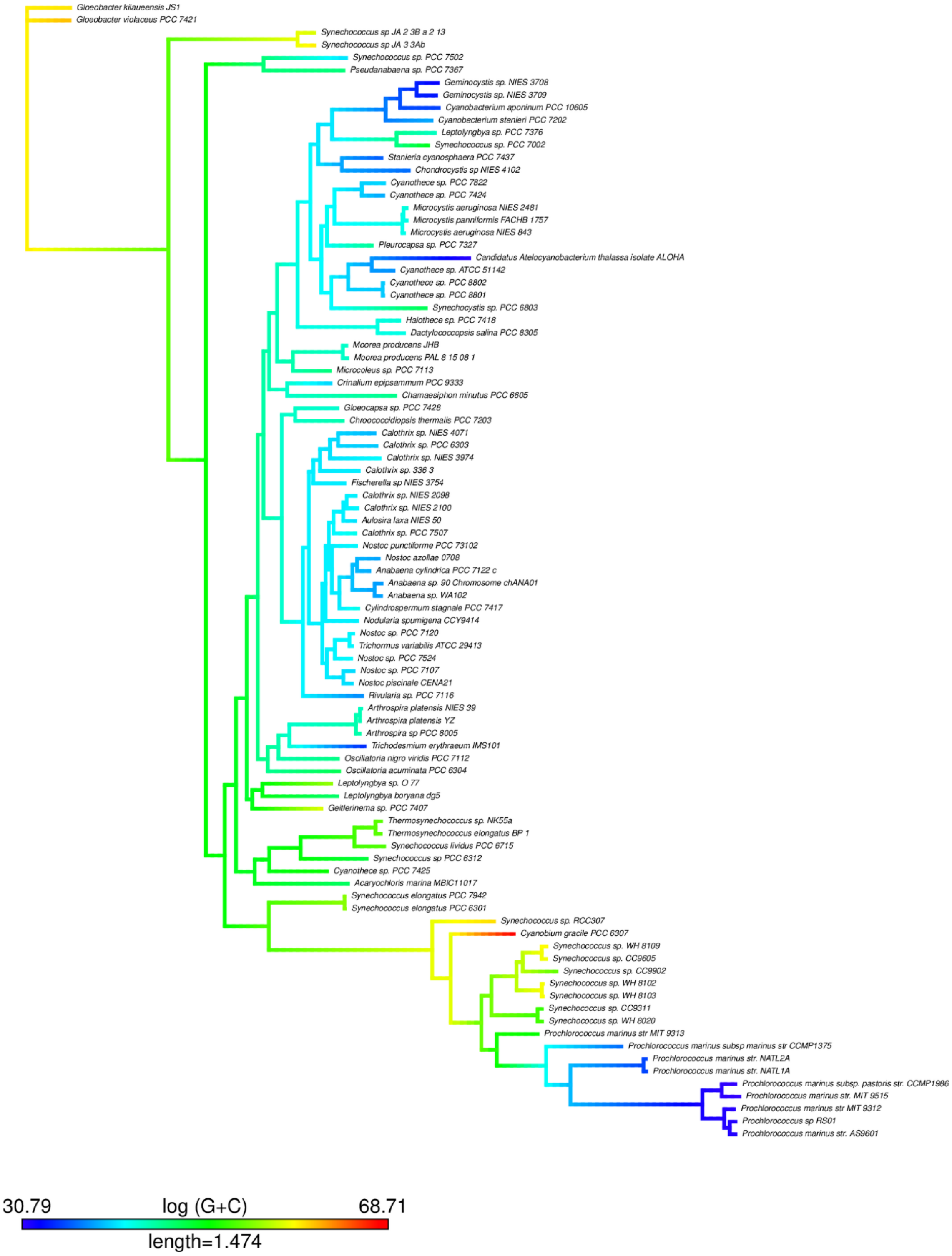
Gloeobacter kilaueensis JS1
Gloeobacter violaceus PCC 7421
Synechococcus sp JA 2 3B a 2 13
Synechococcus sp JA 3 3Ab
Synechococcus sp. PCC 7502
Pseudanabaena sp. PCC 7367
Geminocystis sp. NIES 3708
Geminocystis sp. NIES 3709
Cyanobacterium aponinum PCC 10605
Cyanobacterium stanieri PCC 7202
Leptolyngbya sp. PCC 7376
Synechococcus sp. PCC 7002
Stanieria cyanosphaera PCC 7437
Chondrocystis sp NIES 4102
Cyanothece sp. PCC 7822
Cyanothece sp. PCC 7424
Microcystis aeruginosa NIES 2481
Microcystis panniformis FACHB 1757
Microcystis aeruginosa NIES 843
Pleurocapsa sp. PCC 7327
Candidatus Atelocyanobacterium thalassa isolate ALOHA
Cyanothece sp. ATCC 51142
Cyanothece sp. PCC 8802
Cyanothece sp. PCC 8801
Synechocystis sp. PCC 6803
Halothece sp. PCC 7418
Dactylococcopsis salina PCC 8305
Moorea producens JHB
Moorea producens PAL 8 15 08 1
Microcoleus sp. PCC 7113
Crinalium epipsammum PCC 9333
Chamaesiphon minutus PCC 6605
Gloeocapsa sp. PCC 7428
Chroococcidiopsis thermalis PCC 7203
Calothrix sp. NIES 4071
Calothrix sp. PCC 6303
Calothrix sp. NIES 3974
Calothrix sp. 336 3
Fischerella sp NIES 3754
Calothrix sp. NIES 2098
Calothrix sp. NIES 2100
Aulosira laxa NIES 50
Calothrix sp. PCC 7507
Nostoc punctiforme PCC 73102
Nostoc azollae 0708
Anabaena cylindrica PCC 7122 c
Anabaena sp. 90 Chromosome chANA01
Anabaena sp. WA102
Cylindrospermum stagnale PCC 7417
Nodularia spumigena CCY9414
Nostoc sp. PCC 7120
Trichormus variabilis ATCC 29413
Nostoc sp. PCC 7524
Nostoc sp. PCC 7107
Nostoc piscinale CENA21
Rivularia sp. PCC 7116
Arthrospira platensis NIES 39
Arthrospira platensis YZ
Arthrospira sp PCC 8005
Trichodesmium erythraeum IMS101
Oscillatoria nigro viridis PCC 7112
Oscillatoria acuminata PCC 6304
Leptolyngbya sp. O 77
Leptolyngbya boryana dg5
Geitlerinema sp. PCC 7407
Thermosynechococcus sp. NK55a
Thermosynechococcus elongatus BP 1
Synechococcus lividus PCC 6715
Synechococcus sp PCC 6312
Cyanothece sp. PCC 7425
Acaryochloris marina MBIC11017
Synechococcus elongatus PCC 7942
Synechococcus elongatus PCC 6301
Synechococcus sp. RCC307
Cyanobium gracile PCC 6307
Synechococcus sp. WH 8109
Synechococcus sp. CC9605
Synechococcus sp. CC9902
Synechococcus sp. WH 8102
Synechococcus sp. WH 8103
Synechococcus sp. CC9311
Synechococcus sp. WH 8020
Prochlorococcus marinus str MIT 9313
Prochlorococcus marinus subsp marinus str CCMP1375
Prochlorococcus marinus str. NATL2A
Prochlorococcus marinus str. NATL1A
Prochlorococcus marinus subsp. pastoris str. CCMP1986
Prochlorococcus marinus str. MIT 9515
Prochlorococcus marinus str MIT 9312
Prochlorococcus sp RS01
Prochlorococcus marinus str. AS9601
30.79
log (G+C)
68.71
length=1.474

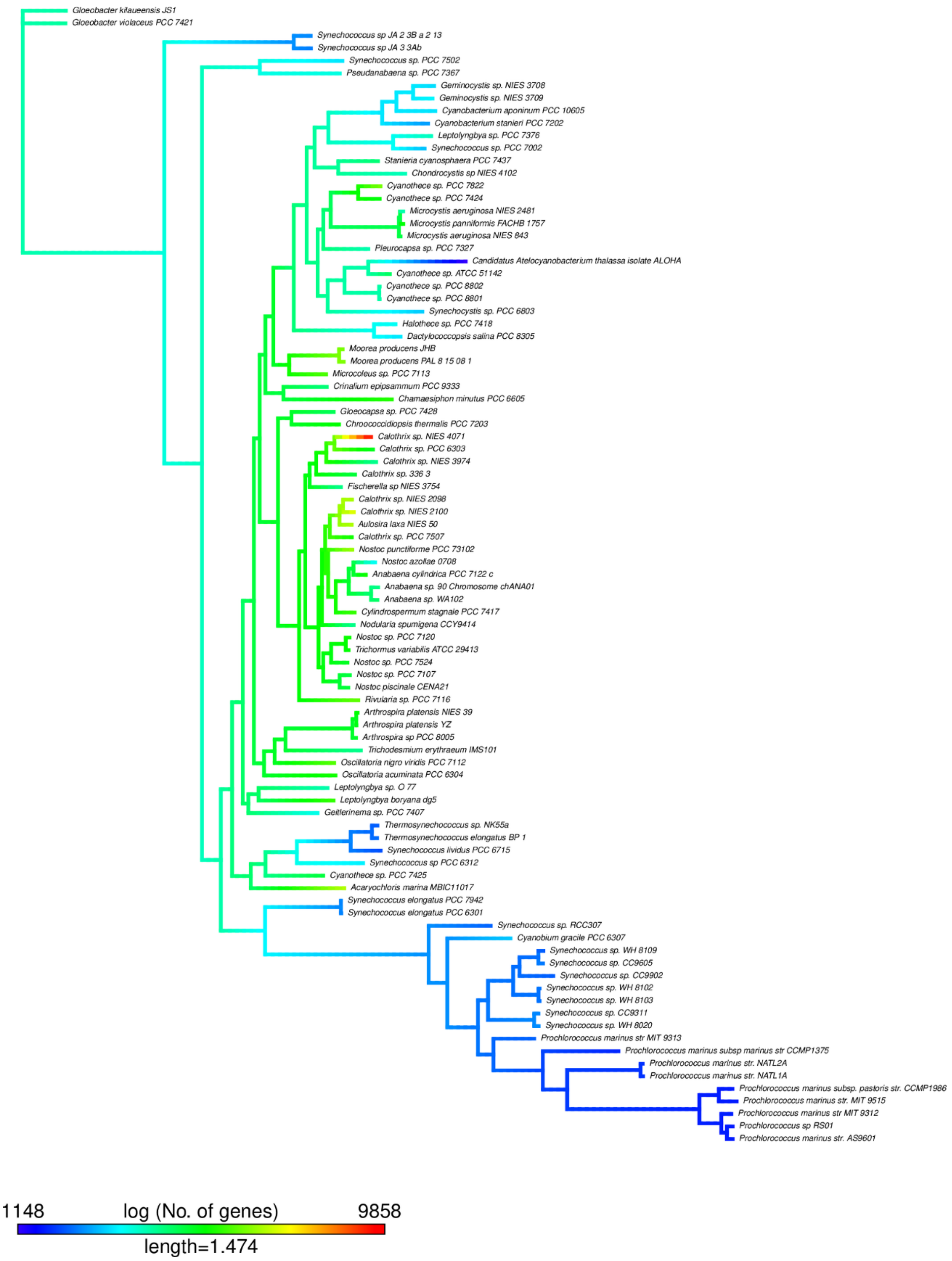

Figure S1. Cyanobacteria tree mapping of the rest of the metrics and parameters.

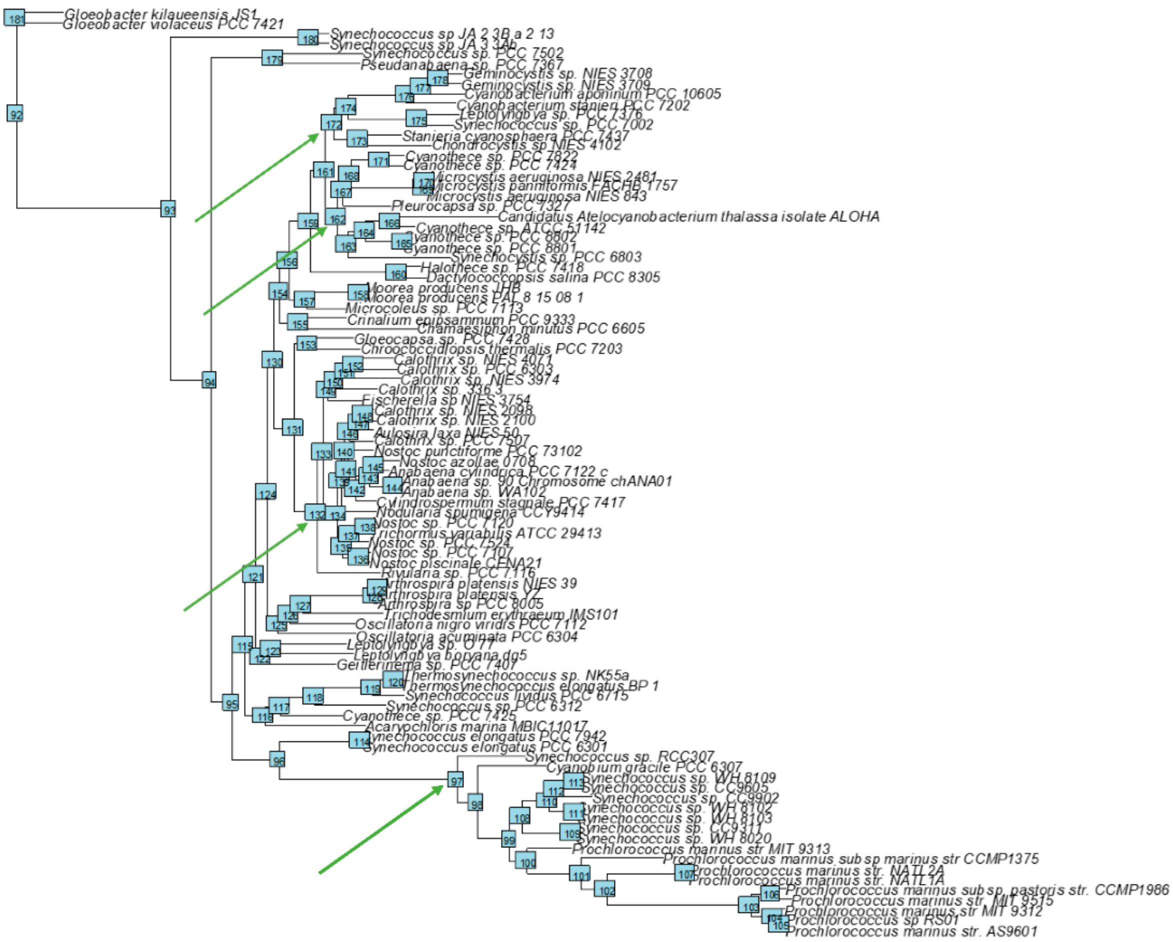

Figure S2. Indication on the phylogenetic tree of the Cyanobacteria the location (green lines) of the four monophyletic clades (97, 132, 162 and 172) where evolutionary trends of metrics and genome parameters were evaluated.

	Phylum (n = 91)		Clade 97 (n = 18)		Clade 132 (n = 22)		Clade 162 (n = 11)		Clade 172 (n = 8)	
**Metrics:**	Slope	P-value	Regression	P-value	Regression	P-value	Regression	P-value	Regression	P-value
SCC	0.14	0.003	0.28	0.023	0.38	0.130	0.22	0.283	0.55	0.062
SCC_SW	0.10	0.013	-0.26	0.008	-0.70	0.016	0.13	0.359	0.36	0.175
SCC_RY	0.23	0.000	0.62	0.000	0.38	0.206	0.02	0.505	0.45	0.121
SCC_KM	0.05	0.158	-0.28	0.007	-0.11	0.337	-0.87	0.006	0.16	0.330
GS	0.09	0.042	0.20	0.096	0.64	0.028	0.53	0.094	0.64	0.038
BB	-0.15	0.004	-0.25	0.053	0.52	0.067	-0.14	0.640	0.02	0.477
**Genome parameters:**										
Genome size	-0.23	0.081	-0.53	0.044	0.46	0.304	-0.90	0.090	-1.36	0.036
%GC	-0.07	0.362	-0.50	0.026	-2.82	0.001	-0.68	0.180	-0.68	0.185
No. of genes	-0.19	0.148	-0.56	0.027	0.36	0.337	-0.59	0.195	-1.52	0.018

Positive trend

Negative trend

Table S2. Ridge regression of genome sequence complexity metrics and genome parameters versus age (distance from the root) in the phylum and the four selected monophyletic clades.

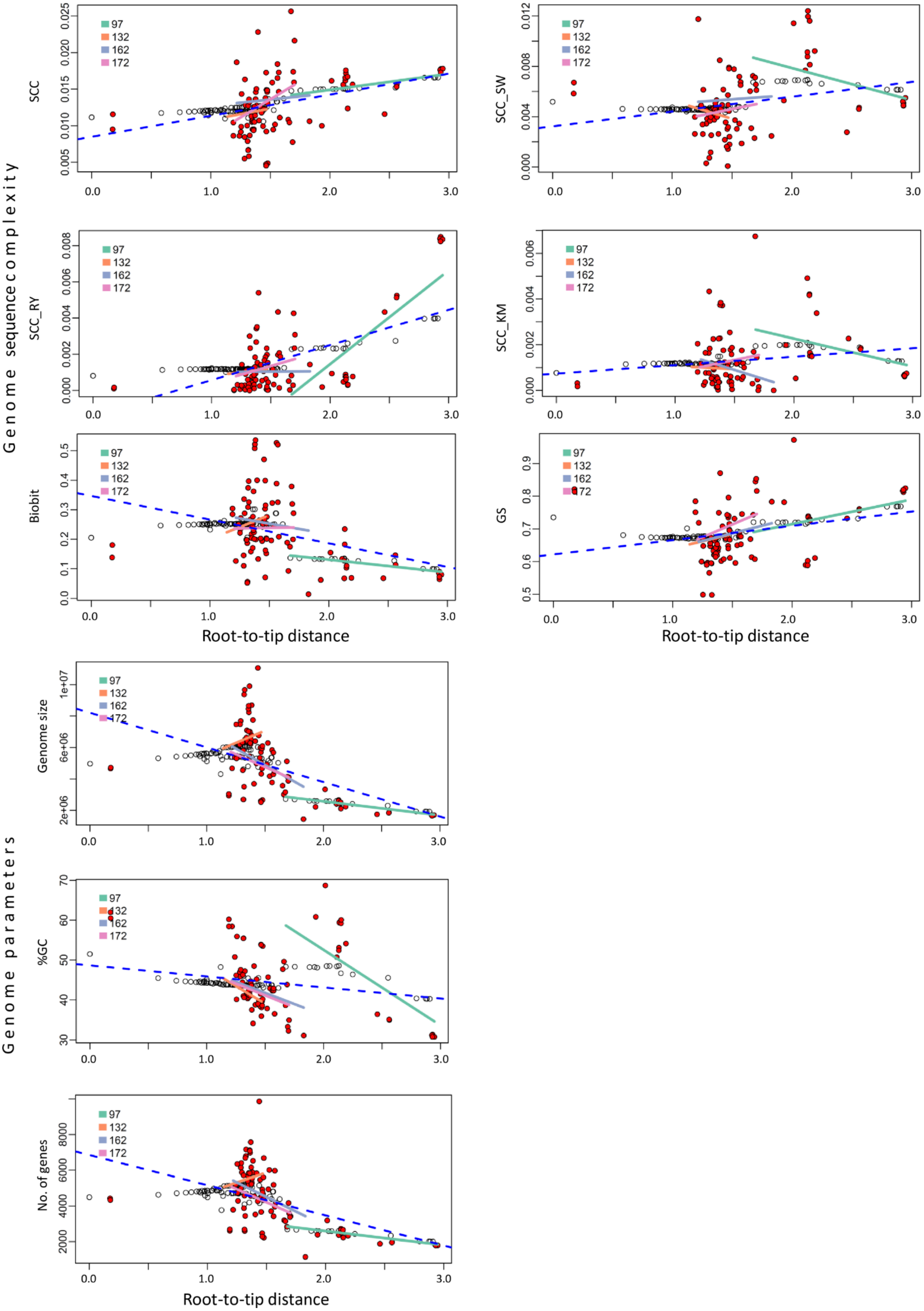
Genome sequence complexity
Genome parameters
SCC
SCC_SW
SCC_RY
SCC_KM
Biobit
GS
Genome size
%GC
No. of genes
97
132
162
172
Root-to-tip distance

Figure S3. Phylogenetic trends of genome sequence complexity metrics and standard genome parameters in the clades 97, 132, 162 and 172. The estimated genomic value for each tip (red dots) or node (black dots) in the phylogenetic tree is regressed against its evolutionary age (i.e. distance from the root). The statistical significance of the regressions are tested against 10,000 slopes obtained under simulated Brownian evolution.

	Phylum		Subclade 97		Subclade 132		Subclade 162		Subclade 172		Mood's Median test *P*-value (Phylum vs. Subclade)			
Metric / Parameter	n	Median	n	Median	n	Median	n	Median	n	Median	Subclade 97	Subclade 132	Subclade 162	Subclade 172
SCC	91	1.24E-02	18	1.64E-02	22	1.14E-02	11	1.47E-02	8	1.35E-02	3.9E-01		3.6E-01	3.3E-01
SCC_{SW}	91	4.48E-03	18	6.59E-03	22	4.29E-03	11	6.51E-03	8	4.65E-03	2.6E-01		3.4E-01	3.1E-01
SCC_{RY}	91	7.35E-04	18	9.12E-04	22	8.19E-04	11	5.46E-04	8	1.35E-03	3.9E-01	4.6E-01		3.3E-01
SCC_{KM}	91	1.00E-03	18	1.59E-03	22	5.96E-04	11	5.93E-04	8	8.04E-04	3.9E-01			
GS	91	6.68E-01	18	7.48E-01	22	6.60E-01	11	6.79E-01	8	7.71E-01	3.9E-01		3.6E-01	3.3E-01
Biobit	91	2.04E-01	18	9.63E-02	22	2.45E-01	11	2.03E-01	8	2.04E-01		4.0E-01		3.3E-01
%GC	91	4.23E+01	18	5.16E+01	22	4.10E+01	11	4.02E+01	8	3.62E+01	3.9E-01			
No. of genes	91	4336	18	2284.5	22	5570.5	11	4371	8	3506.5		4.0E-01	3.6E-01	
Genome Size	91	4934270	18	2168210	22	6524400	11	4934270	8	4132140		4.0E-01	3.6E-01	
Not necessary, as the subclade median is **lower** than the phylum median														

Table S3. Sub-clade test, second proof. Median values in the entire phylum and the four sub-clades. In the protocol adopted here, a subclade drawn from the tail is defined as a monophyletic subset chosen such that the mean fits distribution is greater than the mean of the parent distribution.
